# Prevalence and genotypic distribution of virulence factor genes and antibiotic susceptibility profiles in clinical *Pseudomonas aeruginosa* isolates from a public hospital in Nanyang, China

**DOI:** 10.1128/spectrum.00973-26

**Published:** 2026-06-29

**Authors:** Si-Ping Zhang, Lin-Yu Li, Fei Wang, Bo Niu, Hao-Jie Liu, Guang-Yuan Zhou, Chun-Hui Fan, Fang-Hui Bai, Yong-Xing He

**Affiliations:** 1 Central Laboratory, Nanyang Central Hospital 601574, Nanyang, China; 2 Medical laboratory, Nanyang Central Hospital 601574, Nanyang, China; 3 School of Biological and Chemical Engineering, Nanyang Institute of Technology 154484, Nanyang, China; 4 Ministry of Education Key Laboratory of Cell Activities and Stress Adaptations, School of Life Sciences, Lanzhou University 12426, Lanzhou, China; Yan'an University, Yan'an, Shaanxi, China

**Keywords:** *Pseudomonas aeruginosa*, multidrug resistant, antibiotic susceptibility profile, virulence factor genes, genotype distribution

## Abstract

*Pseudomonas aeruginosa* remains a critical global health concern, particularly in low- and middle-income countries, owing to its high carriage of diverse virulence factor genes (VFGs) and multidrug resistance. Here, we analyzed the prevalence and genotype distribution of VFGs in *P. aeruginosa* isolated from Nanyang Central Hospital (NYCH), China, along with their antibiotic susceptibility profiles. The study involved 79 multidrug-resistant (MDR) and 96 non-MDR (nMDR) strains and the following 11 key VFGs (*nan1*, *pilA*, *aprA*, *phzS*, *plcH*, *toxA*, *lasB*, *exoS*, *exoU*, *exoT*, *exoY*). Antibiogram profiling showed near-universal resistance to at least one antibiotic class. MDR strains exhibited 23 distinct antibiotic resistance patterns, compared with 13 patterns in nMDR strains. A moderate negative correlation was observed between β-lactams/β-lactamase inhibitors and fluoroquinolone resistance. Moreover, statistical analysis revealed that the *phzS* gene displayed the most significant prevalence difference between MDR and nMDR subgroups (*P* < 0.001). Finally, 28 distinct genotypes were identified in nMDR isolates, and 26 genotypes appeared in MDR strains. There was a statistically significant correlation found between the presence of genotype XX (r = 0.481, *P* < 0.001) and genotype XXVIII (r = −0.320, *P* < 0.001) in two subgroups. Collectively, our findings provide data support for understanding the pathogenic mechanism of local *P. aeruginosa*, and are of great significance for guiding clinical anti-infection treatment and infection control.

**IMPORTANCE:**

*Pseudomonas aeruginosa* is an opportunistic pathogen notorious for causing difficult-to-treat infections, particularly among hospitalized patients in resource-limited healthcare settings. This study analyzed clinical isolates from a major hospital in China to investigate the prevalence and genotypic distribution of virulence factor genes (VFGs) and antibiotic susceptibility profiles of *P. aeruginosa*. Our results showed that multidrug-resistant (MDR) strains displayed more complex and diverse resistance profiles than non-MDR (nMDR) strains. Both MDR and nMDR isolates exhibited high genotypic diversity. Notably, a statistically significant association was observed between genotype XX and genotype XXVIII across the two subgroups. These findings elucidate the molecular basis underlying the variable treatment difficulty of *P. aeruginosa* infections and offer valuable insights for guiding empirical antibiotic selection. Characterizing local bacterial features, including antibiotic susceptibility profiles and the distribution of clinically relevant virulence factors, may improve understanding of regional resistance trends and support antimicrobial stewardship and infection control efforts.

## INTRODUCTION

*Pseudomonas aeruginosa* (*P. aeruginosa*), as a Gram-negative opportunistic pathogen, frequently colonizes immunocompromised patients and causes severe acute and chronic infections (1, 2). The emergence of multidrug-resistant (MDR), extensively drug-resistant (XDR), pan-drug-resistant, and carbapenem-resistant *P. aeruginosa* (CRPA) strains has raised global concern, with frequent outbreaks reported in critical care units, surgical wards, and burn units (3, 4). Recent epidemiological data from the European network for intensive care unit (ICU)-related respiratory infections secondary analysis indicates that *P. aeruginosa* was detected in 14.5% of nosocomial respiratory infection specimens among ICU patients (5). Recognizing the threat posed by *P. aeruginosa*, the World Health Organization classified *P. aeruginosa* as a critical priority pathogen requiring urgent development of new antibiotics (6–8). Besides, it is also recognized as a core member of the six “ESKAPE” pathogens (9), underscoring the urgent need for enhanced surveillance, early detection, infection control measures, and the development of novel therapeutic strategies against *P. aeruginosa*.

The high incidence and mortality of *P. aeruginosa* infections arise from its multidrug resistance and an array of virulence factor genes (VFGs) in its versatile genome (10, 11). This bacterium displays intrinsic resistance to multiple antibiotic classes (e.g., β-lactams, fluoroquinolones, and aminoglycosides) and readily develops MDR or XDR strains via genetic mutations, horizontal gene transfer of resistance determinants (e.g., genes encoding extended-spectrum β-lactamases and carbapenemases), and robust biofilm formation (3, 12–16). Its pathogenicity is driven by VFGs that collectively facilitate host tissue adhesion, invasion, immune evasion, and direct tissue damage, severely limiting therapeutic options and contributing to poor clinical outcomes in vulnerable patients (17, 18).

Examples of key VFGs include *nan1*, *pilA*, *aprA*, *phzS*, *plcH*, *toxA*, *lasB*, *exoS*, *exoU*, *exoT*, and *exoY*. These enable mucosal adhesion (e.g., via neuraminidase Nan1 and type IV pilus subunit PilA) (19–23), tissue destruction (e.g., zinc metalloproteinase LasB and AprA, hemolytic phospholipase C PlcH) (24–29), immune evasion (e.g., ExoS-mediated disruption of host cytoskeletal dynamics and phagocytosis inhibition) (30–32), cell lysis and cytotoxicity (e.g., phospholipase A2 ExoU) (33, 34), inhibition of host protein synthesis (e.g., exogenous toxin A ToxA) (23, 35), and oxidative damage (via pyocyanin biosynthesis involving the PhzS pathway) (35), severely limiting treatment efficacy of *P. aeruginosa* infections. Although the precise roles of ExoT and ExoY in *P. aeruginosa* are less well characterized, current evidence indicates that ExoT modulates host cell cycle progression (30, 36), whereas ExoY primarily functions as an adenylyl cyclase, elevating intracellular cAMP levels in target cells and disrupting normal signaling (37, 38). Antimicrobial resistance and virulence traits often co-evolve in high-risk *P. aeruginosa* clones, rather than being mutually exclusive, resulting in “superbug” strains with heightened pathogenicity and therapeutic challenges (14, 39). For example, the most representative high-risk clone ST235 frequently harbors both the *exoU* virulence gene and over 60 distinct β-lactamase variants, contributing to therapeutic failure and elevated patient mortality in invasive *P. aeruginosa* infections (14). A comprehensive understanding of the antibiotic susceptibility profiles and virulence factors of *P. aeruginosa* isolates is essential for developing targeted interventions against *P. aeruginosa* infections and improving clinical prognosis.

The dissemination of MDR *P. aeruginosa* strains harboring VFGs poses a major global public health challenge, particularly in low- and middle-income countries (LMICs) across Africa and Asia, contributing significantly to increase the risk of hospital-acquired infections and the economic burden (40). Furthermore, the antibiotic susceptibility profiles and distribution of key VFGs in *P. aeruginosa* exhibit pronounced setting- and geography-specific patterns (41, 42), as evidenced by significant worldwide variation in the detection rates of the genes, including *toxA* (43, 44), *pilA* (44, 45), and *exoU* (44, 46). Updated information on antibiotic resistance patterns and virulence features in MDR strains remains limited in many hospital settings across LMICs. In this study, we compared the prevalence and genotype distribution of VFGs in MDR and non-MDR (nMDR) strains of *P. aeruginosa* from Nanyang Central Hospital (NYCH). The presence of 11 VFGs (*nan1*, *pilA*, *aprA*, *phzS*, *plcH*, *toxA*, *lasB*, *exoS*, *exoU*, *exoT*, *exoY*) of these strains was investigated. In addition, the antibiotic resistance patterns of these isolates were also analyzed.

## RESULTS

### The origin, prevalence, and antimicrobial resistance of *P. aeruginosa* isolates

**Fig 1:**
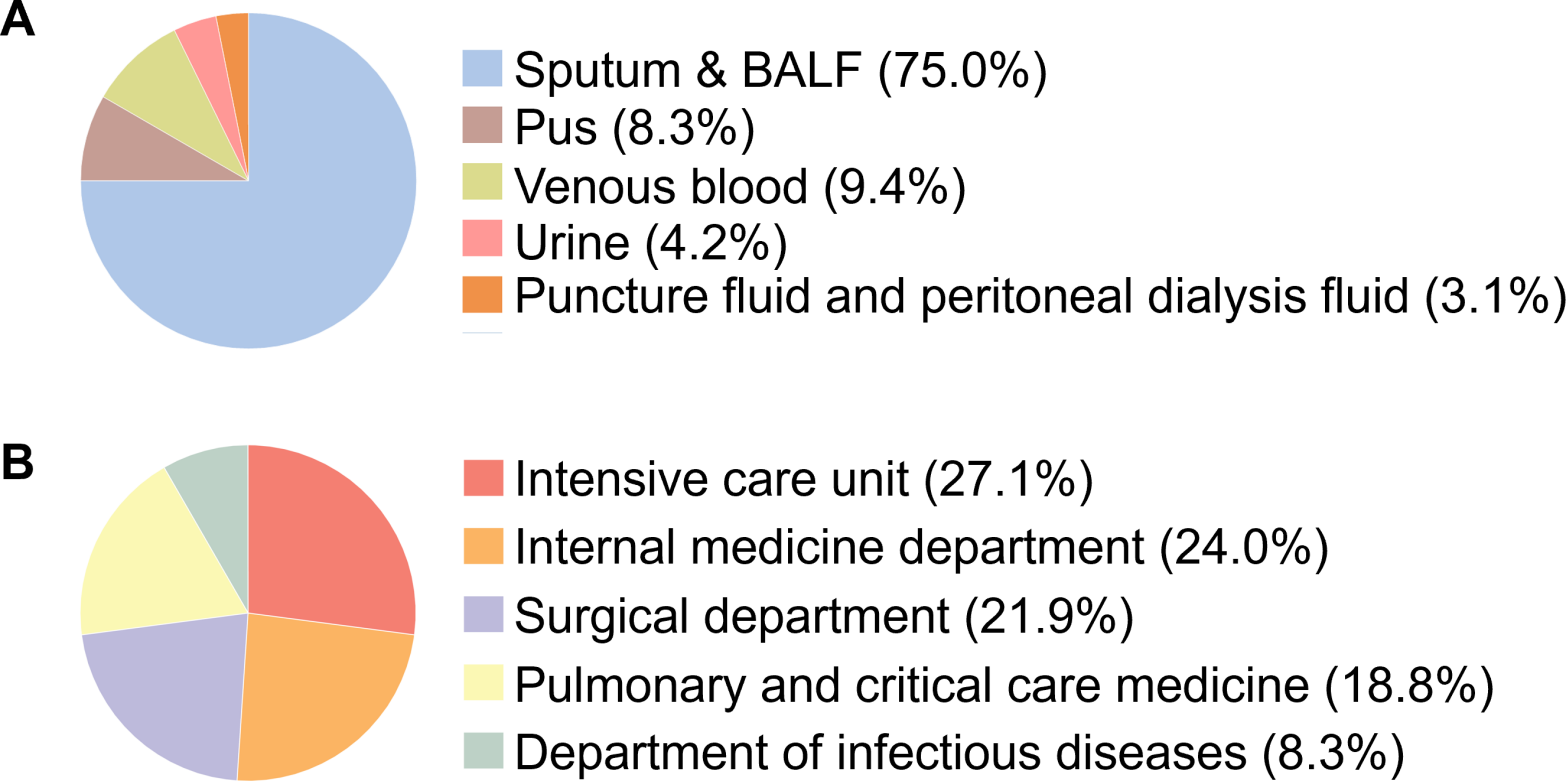
The specimen types (**A**) and departments (**B**) of *P. aeruginosa* included in this study (*n* = 175).

The overall antibiogram profiles of the isolated *P. aeruginosa* strains are presented in Table S2. Based on the antimicrobial susceptibility testing (AST) results detailed therein, this study included a total of 96 nMDR and 79 MDR clinical isolates of *P. aeruginosa*. The origins (specimen types and unit origins) of the nMDR and MDR isolates are shown in Fig. 1, while Fig. S1 and S2 provide separate, detailed presentations of the nMDR and MDR isolate distributions, respectively.

Antimicrobial resistance rates among the 175 isolates were as follows: β-lactams/β-lactamase inhibitors (BL/BLI) (*n* = 101, 57.7%), Fluoroquinolones (FQs) (*n* = 111, 63.4%), Monobactams (MBs) (*n* = 99, 56.6%), Cephalosporins (CEPs) (*n* = 50, 28.6%), Carbapenems (CBPs) (*n* = 46, 26.3%), Penicillins (PCNs) (*n* = 26, 14.9%), and Aminoglycosides (AGs) (*n* = 14, 8.0%). No resistance to polymyxins was observed in any isolate (Table S3). Nearly all isolates (*n* = 174, 99.4%) exhibited resistance to at least one antibiotic class. Moreover, 97.5% of the MDR isolates were resistant to BL/BLI (Table S2). The correlation analysis of resistance patterns revealed a moderate negative correlation between MBs and FQs (r = −0.474, *P* < 0.001). Moderate positive correlations were observed between CEPs and PCNs (r = 0.554, *P* < 0.001), CEPs and BL/BLI (r = 0.465, *P* < 0.001), and CEPs and AGs (r = 0.420, *P* < 0.001). Weak positive correlations were found in six additional pairs: BL/BLI and PCNs (r = 0.325, *P* < 0.001), BL/BLI and MBs (r = 0.370, *P* < 0.001), PCNs and FQs (r = 0.284, *P* < 0.001), CEPs and CBPs (r = 0.255, *P* = 0.001), AGs and CBPs (r = 0.302, *P* < 0.001), CBPs and BL/BLI (r = 0.327, *P* < 0.001) (Table S4).

In the group of MDR strains, the only moderate positive correlation observed was that of the CEPs and PCNs (r = 0.451, *P* < 0.001). Weak positive relationships were observed between the AGs and CEPs (r = 0.366, *P* = 0.001), PCNs and FQs (r = 0.305, *P* = 0.006), AGs and CBPs (r = 0.233, *P* = 0.038), MBs and BL/BLI (r = 0.259, *P* = 0.021). Weak negative correlations were noted between AGs and BL/BLI (r = −0.363, *P* = 0.001), AGs and MBs (r = −0.257, *P* = 0.022) (Table S5). In the group of nMDR strains, bivariate analysis exhibited that the MBs had a weak positive correlation with BL/BLI (r = 0.267, *P* = 0.009). In contrast, a strong negative correlation was found between MBs and FQs (r = −0.877, *P* < 0.001), accompanied by 2 weak negative correlations between FQs and BL/BLI (r = −0.361, *P* < 0.001), CBPs and MBs (r = −0.228, *P* = 0.026) (Table S6).

### Antibiotic resistance patterns of *P. aeruginosa* isolates

Among the 175 *P. aeruginosa* isolates, a total of 36 distinct antibiotic resistance patterns were identified (Table S7). In the subgroup of 79 MDR isolates, 23 different resistance patterns were observed, with the combination of resistance to BL/BLI, FQs, and MBs being the most frequent (*n* = 12; 15.2%). Two isolates showed resistance to seven antimicrobial classes (PCNs, BL/BLI, CEPs, AGs, FQs, MBs, and CBPs). Additionally, 14 isolates exhibited resistance to more than six antibiotic classes, and 40 CRPA isolates were collected. In contrast, the 96 nMDR isolates displayed 13 distinct resistance patterns. Among these isolates, resistance was most frequently observed to FQs (38.5%), followed by MBs (25.0%). Table S7 summarizes the distribution of the antibiotic resistance patterns of *P. aeruginosa* collection.

### The prevalence of VFGs amongst MDR and nMDR strains

**Fig 2:**
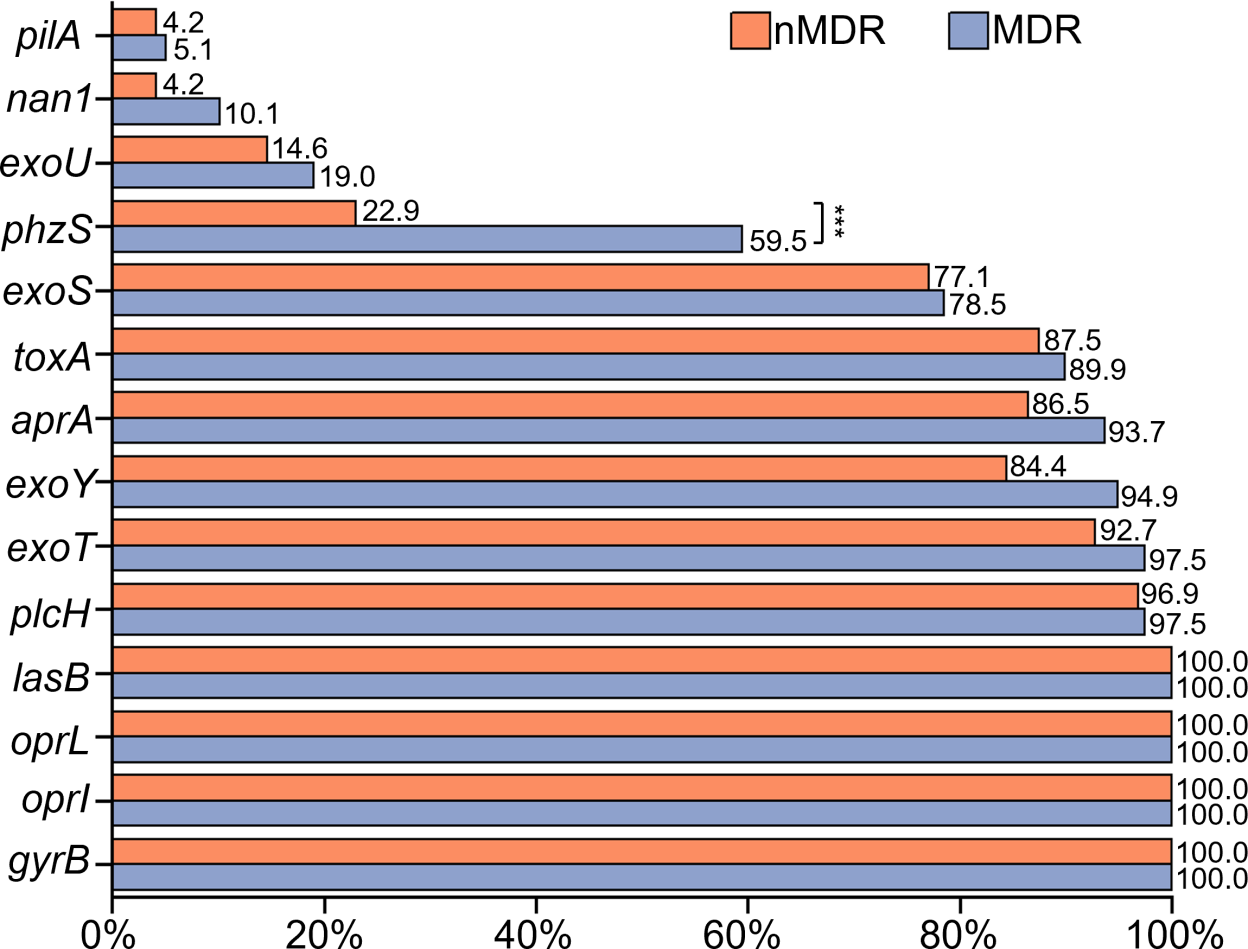
Prevalence of detected VFGs of *P. aeruginosa* included in the study according to the AST results (nMDR, *n* = 96 vs MDR, *n* = 79).

Gene-specific simplex polymerase chain reaction (PCR) was employed to screen for VFGs in 175 *P. aeruginosa* isolates. Two outer membrane lipoprotein genes, *oprL* and *oprI* (47), together with the housekeeping gene *gyrB* (encoding DNA gyrase subunit B) (48, 49), were further used to confirm *P. aeruginosa*. One hundred percent of isolates were positive for the *oprL*, *oprI*, and *gyrB* genes. The analysis revealed a variable distribution of VFGs. The incidence of VFGs was as follows: the *phzS* gene was detected in 39.4% of strains, while *toxA*, *aprA*, and *plcH* showed high frequencies at 88.6%, 89.7%, and 97.1%, respectively. The *lasB* gene was detected in all the tested strains, whereas the *pilA* and *nan1* genes exhibited the lowest detection rates, at only 4.6% and 6.9%, respectively. In the type III secretion system effector gene family, *exoT* was the most common (94.9%), followed by *exoY* (89.1%), *exoS* (77.7%), and *exoU* (16.6%) (Table S8). The gene prevalence pattern in the overall collection is similar to that observed when MDR and nMDR isolates are analyzed separately (Fig. 2). The prevalence of the *phzS* gene displayed the most significant difference between MDR and nMDR subgroups of strains (*P* < 0.001) (Fig. 2). The gel electrophoresis results of all tested genes in representative samples are presented in Fig. S3 and S4. The detailed incidence of the detected VFGs amongst the examined *P. aeruginosa* is provided in Table S9 for the MDR strains and Table S10 for the nMDR isolates.

### Prevalence of the observed genotypes amongst MDR and nMDR strains

**Fig 3:**
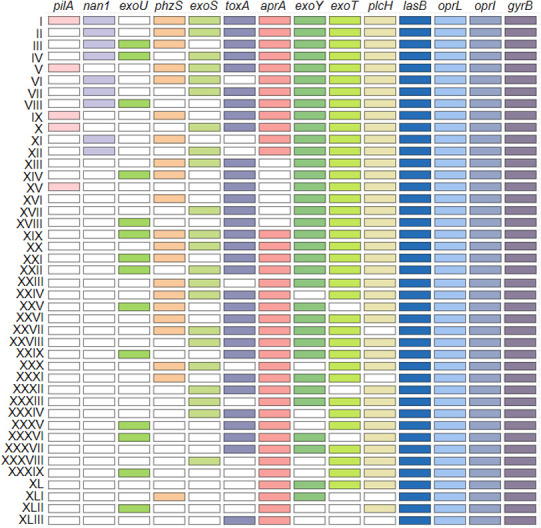
Characteristics of the genotypes detected amongst examined *P. aeruginosa* strains (nMDR, *n* = 96 vs MDR, *n* = 79). Dark color indicates the presence of the gene, white color indicates its absence.

Assessment of the detected VFGs in this study revealed considerable genotypic diversity among the clinical *P. aeruginosa* isolates. A total of 43 distinct genotypes (named I–XLIII) were identified across all examined strains, with their prevalence and distribution illustrated in Fig. 3 and detailed in Table 1. The most prevalent genotype, numbered XXVIII, including the genes *aprA*, *plcH*, *exoS*, *exoT*, *exoY*, *toxA*, *lasB*, *oprL*, *oprI*, and *gyrB*, was observed among 50 of the isolates. Of note, the highest number of VFGs co-occurring in a single isolate was 13, observed in 1 MDR strain.

**TABLE 1:** Distribution of genotypes detected amongst examined MDR and nMDR *P. aeruginosa* strains

Genotype	Number of MDR isolates	Number of nMDR isolates
I	1 (1.3%)	0 (0.0%)
II	3 (3.8%)	0 (0.0%)
III	1 (1.3%)	0 (0.0%)
IV	1 (1.3%)	0 (0.0%)
V	3 (3.8%)	0 (0.0%)
VI	0 (0.0%)	1 (1.0%)
VII	0 (0.0%)	2 (2.1%)
VIII	0 (0.0%)	1 (1.0%)
IX	0 (0.0%)	1 (1.0%)
X	0 (0.0%)	2 (2.1%)
XI	1 (1.3%)	0 (0.0%)
XII	1 (1.3%)	0 (0.0%)
XIII	1 (1.3%)	5 (5.2%)
XIV	0 (0.0%)	1 (1.0%)
XV	0 (0.0%)	1 (1.0%)
XVI	0 (0.0%)	1 (1.0%)
XVII	3 (3.8%)	5 (5.2%)
XVIII	1 (1.3%)	0 (0.0%)
XIX	3 (3.8%)	0 (0.0%)
XX	28 (35.4%)	0 (0.0%)
XXI	2 (2.5%)	0 (0.0%)
XXII	1 (1.3%)	0 (0.0%)
XXIII	0 (0.0%)	1 (1.0%)
XXIV	1 (1.3%)	3 (3.1%)
XXV	0 (0.0%)	1 (1.0%)
XXVI	2 (2.5%)	2 (2.1%)
XXVII	1 (1.3%)	1 (1.0%)
XXVIII	10 (12.7%)	40 (41.7%)
XXIX	5 (6.3%)	8 (8.3%)
XXX	0 (0.0%)	3 (3.1%)
XXXI	0 (0.0%)	1 (1.0%)
XXXII	1 (1.3%)	3 (3.1%)
XXXIII	3 (3.8%)	1 (1.0%)
XXXIV	0 (0.0%)	3 (3.1%)
XXXV	0 (0.0%)	1 (1.0%)
XXXVI	0 (0.0%)	1 (1.0%)
XXXVII	2 (2.5%)	1 (1.0%)
XXXVIII	1 (1.3%)	4 (4.2%)
XXXIX	1 (1.3%)	0 (0.0%)
XL	1 (1.3%)	0 (0.0%)
XLI	0 (0.0%)	1 (1.0%)
XLII	0 (0.0%)	1 (1.0%)
XXLIII	1 (1.3%)	0 (0.0%)

Among the 79 MDR isolates, 26 distinct genotypes were identified. The most prevalent genotype, designated XX, harboring the genes *aprA*, *phzS*, *plcH*, *exoS*, *exoT*, *exoY*, *toxA*, *lasB*, *oprL*, *oprI*, and *gyrB*, was detected in 28 isolates (35.4%). Genotype XXVIII was the second most common, occurring in 10 isolates (12.7%). Genotypes XXVI, XXVII, XXXIII, and XXXVII showed higher prevalence in MDR isolates compared with nMDR isolates. In the group of 96 nMDR isolates, 28 distinct genotypes were observed. The dominant genotype was XXVIII, which was detected in 40 isolates (41.7%). Genotype XXIX ranked second, occurring in eight isolates (8.3%). All other genotypes were relatively rare in both MDR and nMDR groups (Table 1). Overall, the presence of specific VFGs did not predetermine multidrug resistance among the isolates. There was a statistically significant correlation found between the presence of genotype XX (r = 0.481, *P* < 0.001) and genotype XXVIII (r = −0.320, *P* < 0.001) in the nMDR or MDR groups of strains.

In the group of MDR strains, correlation analysis revealed one moderate negative correlation between *exoS* and *exoU* (r = −0.532, *P* < 0.001). In contrast, a moderate positive correlation was found between *plcH* and *exoT* (r = 0.487, *P* < 0.001). Five additional weak positive correlations were identified: *exoS* and *phzS* (r = 0.258, *P* = 0.022), *phzS* and *toxA* (r = 0.321, *P* = 0.004), *exoY* and *plcH* (r = 0.330, *P* = 0.003), *exoT* and *exoY* (r = 0.330, *P* = 0.003), *exoY* and *toxA* (r = 0.305, *P* = 0.006) (Table 2). In the group of nMDR strains, a strong negative correlation was found between *exoS* and *exoU* (r = −0.758, *P* < 0.001). Weak negative correlations were noted between *phzS* and *aprA* (r = −0.290, *P* = 0.004), *phzS* and *plcH* (r = −0.329, *P* = 0.001), *phzS* and *toxA* (r = −0.242, *P* = 0.018), *exoT* and *exoU* (r = −0.225, *P* = 0.028). Additionally, a moderate positive significant correlation existed between *toxA* and *exoY* (r = 0.531, *P* < 0.001), and a weak positive correlation between *exoT* and *exoS* (r = 0.228, *P* = 0.025) (Table 3).

**TABLE 2:** The Spearman's rank correlation coefficients for the particular gene pair in 79 MDR samples^*a*^

MDR (*n* = 79)		nan1	pilA	aprA	phzS	plcH	exoS	exoU	exoT	exoY	toxA
nan1	r	1.000	0.13	0.082	0.08	0.051	−0.052	0.074	0.051	0.073	−0.19
	*P*		0.255	0.474	0.489	0.658	0.654	0.517	0.658	0.525	0.096
pilA	r	0.13	1.000	0.06	0.191	0.037	0.121	−0.112	0.037	0.053	0.078
	*P*	0.255		0.599	0.093	0.745	0.288	0.327	0.745	0.641	0.497
aprA	r	0.082	0.06	1.000	0.209	−0.042	−0.01	−0.007	−0.042	−0.06	−0.087
	*P*	0.474	0.599		0.064	0.714	0.933	0.953	0.714	0.599	0.444
phzS	r	0.08	0.191	0.209	1.000	0.031	0.258*	−0.192	0.195	0.162	0.321*
	*P*	0.489	0.093	0.064		0.785	0.022	0.090	0.085	0.153	0.004
plcH	r	0.051	0.037	−0.042	0.031	1.000	0.112	0.078	0.487**	0.330*	−0.054
	*P*	0.658	0.745	0.714	0.785		0.327	0.494	<0.001	0.003	0.636
exoS	r	−0.052	0.121	−0.01	0.258*	0.112	1.000	−0.532**	0.112	0.16	0.131
	*P*	0.654	0.288	0.933	0.022	0.327		<0.001	0.327	0.159	0.252
exoU	r	0.074	−0.112	−0.007	−0.192	0.078	−0.532**	1.000	0.078	−0.035	0.056
	*P*	0.517	0.327	0.953	0.090	0.494	<0.001		0.494	0.757	0.627
exoT	r	0.051	0.037	−0.042	0.195	0.487**	0.112	0.078	1.000	0.330*	−0.054
	*P*	0.658	0.745	0.714	0.085	<0.001	0.327	0.494		0.003	0.636
exoY	r	0.073	0.053	−0.06	0.162	0.330*	0.16	−0.035	0.330*	1.000	0.305*
	*P*	0.525	0.641	0.599	0.153	0.003	0.159	0.757	0.003		0.006
toxA	r	−0.19	0.078	−0.087	0.321*	−0.054	0.131	0.056	−0.054	0.305*	1.000
	*P*	0.096	0.497	0.444	0.004	0.636	0.252	0.627	0.636	0.006	

^*a*^ Types of correlation: r > 0, positive correlation; r = 0, lack of correlation; r < 0, negative correlation; the strength of relation: <0.2, lack of relation; 0.2–0.4, weak (*); 0.4–0.7, moderate (**); 0.7–0.9, quite strong (***); >0.9, very strong (****).

**TABLE 3:** The Spearman's rank correlation coefficients for the particular gene pair in 96 nMDR samples^*a*^

nMDR (*n* = 96)		nan1	pilA	aprA	phzS	plcH	exoS	exoU	exoT	exoY	toxA
nan1	r	1.000	−0.043	0.083	0.009	0.037	−0.01	0.062	0.058	0.09	−0.079
	*P*		0.674	0.424	0.930	0.717	0.920	0.551	0.571	0.385	0.445
pilA	r	−0.043	1.000	−0.07	0.009	0.037	−0.134	−0.086	0.058	0.09	0.079
	*P*	0.674		0.499	0.930	0.717	0.192	0.404	0.571	0.385	0.445
aprA	r	0.083	−0.07	1.000	−0.290*	−0.071	0.002	0.077	−0.111	−0.17	−0.15
	*P*	0.424	0.499		0.004	0.491	0.988	0.454	0.282	0.097	0.146
phzS	r	0.009	0.009	−0.290*	1.000	−0.329*	−0.189	−0.073	−0.036	−0.173	−0.242*
	*P*	0.930	0.930	0.004		0.001	0.067	0.480	0.728	0.094	0.018
plcH	r	0.037	0.037	−0.071	−0.329*	1.000	0.187	0.074	0.18	−0.077	0.113
	*P*	0.717	0.717	0.491	0.001		0.068	0.472	0.079	0.454	0.272
exoS	r	−0.01	−0.134	0.002	−0.189	0.187	1.000	−0.758***	0.228*	−0.098	−0.056
	*P*	0.920	0.192	0.988	0.067	0.068		<0.001	0.025	0.342	0.587
exoU	r	0.062	−0.086	0.077	−0.073	0.074	−0.758***	1.000	−0.225*	0.015	0.067
	*P*	0.551	0.404	0.454	0.480	0.472	<0.001		0.028	0.883	0.517
exoT	r	0.058	0.058	−0.111	−0.036	0.18	0.228*	−0.225*	1.000	−0.01	0.136
	*P*	0.571	0.571	0.282	0.728	0.079	0.025	0.028		0.920	0.185
exoY	r	0.09	0.09	−0.17	−0.173	−0.077	−0.098	0.015	−0.01	1.000	0.531**
	*P*	0.385	0.385	0.097	0.094	0.454	0.342	0.883	0.920		<0.001
toxA	r	−0.079	0.079	−0.15	−0.242*	0.113	−0.056	0.067	0.136	0.531**	1.000
	*P*	0.445	0.445	0.146	0.018	0.272	0.587	0.517	0.185	<0.001	

^*a*^ Types of correlation: r > 0, positive correlation; r = 0, lack of correlation; r < 0, negative correlation; the strength of relation: <0.2, lack of relation; 0.2–0.4, weak (*); 0.4–0.7, moderate (**); 0.7–0.9, quite strong (***); >0.9, very strong (****).

## DISCUSSION

*P. aeruginosa* is a major global pathogen responsible for hospital-acquired infections and exhibits multidrug resistance alongside the co-evolution of key VFGs, presenting a serious challenge to public health (50–52). This study conducted a comprehensive molecular screening of multiple VFGs and antimicrobial susceptibility profiles in a large collection of clinical isolates (175 samples), aiming to systematically monitor the variability of virulence genotypes in *P. aeruginosa*, thereby providing new insights to inform clinical surveillance and infection control strategies. Of these isolates, 74.9% were obtained from sputum and bronchoalveolar lavage fluid.

The VFGs were selected based on previous studies (30, 43–45). These genes encode a diverse range of factors with distinct biological activities, including components of the type III secretion system (30, 53–55), major enzymes (24–29), as well as various structural and adhesion-related factors (19–23). Both well-characterized genes and some less extensively studied ones were included. The identification of *P. aeruginosa* isolates from clinical samples by MS was further confirmed by PCR detection of *oprL*, *oprI* (47), and *gyrB* genes (48, 49). Previous studies have shown that *oprL* is nearly universally present (often 95%–100%) across diverse clinical and environmental settings, whereas *oprI* exhibits high but slightly more variable detection rates (56). Consistent with these findings, all isolates in the present study were positive for *oprL*, *oprI*, and *gyrB*, confirming their reliability as molecular markers for species identification.

To our knowledge, this represents one of the largest studies to date in terms of the number of VFGs simultaneously investigated. The frequency distribution of VFGs varied substantially among isolates, resulting in a remarkably high number of distinct genotypes, as anticipated. Of note, PCR-based detection may be affected by sequence variation within primer-binding regions, potentially resulting in false-negative results and underestimation of gene prevalence (57, 58). Although the primers have been widely validated and applied in previous studies (30, 43–45, 59–63), the presence of undetected variants cannot be excluded. Moreover, while PCR-based detection confirms gene presence, it does not provide information on gene functionality or expression. Future studies incorporating whole-genome sequencing (WGS) and phenotypic assessment of resistance mechanisms will be essential to evaluate sequence variations, potential inactivating mutations, and their impact on the pathogenicity and clinical behavior of *P. aeruginosa*.

*P. aeruginosa* infections are difficult to treat due to its intrinsic resistance mechanisms and remarkable ability to acquire resistance to multiple antibiotic classes (10, 43, 64, 65). In this study, we identified 79 MDR and 14 XDR *P. aeruginosa* isolates, underscoring a worrisome level of resistance to commonly used antipseudomonal agents. The XDR isolates were predominantly from surgical wards (50.0%), followed by ICUs (28.6%) and internal medicine departments (21.4%) (Tables S11 and S12). XDR *P. aeruginosa* infections are linked to elevated mortality, extended hospital stays, and markedly increased healthcare costs, especially when treatment options are severely restricted (66, 67). Compared to recent studies, the antimicrobial resistance profile observed in this study reflects both global trends and regional variation in *P. aeruginosa*. The high resistance rates to FQs (63.4%) and BL/BLI (57.7%) are consistent with reports from meta-analyses conducted in Asia and Africa (68), although they remain lower than those reported in EU/EEA countries, including Poland (69). Notably, resistance rates to CEPs (28.6%) and PCNs (14.9%) were lower than those described in meta-analyses from Asia and Africa (68). These differences may be attributable to variations in local antimicrobial prescribing practices, infection control measures, and the prevalence of high-risk resistant clones. Nevertheless, the observed CBP resistance rate (26.3%) remains clinically significant and aligns with intermediate levels reported in international surveillance programs, underscoring the ongoing threat posed by CRPA (70, 71). The high susceptibility rate to AGs shown in our study (92.0%), consistent with most previous findings (41, 42, 72), may reflect their relatively judicious use due to concerns regarding toxicity. Meanwhile, no resistance to polymyxins was detected, supporting their continued role as last-line agents against MDR organisms (42, 73). These findings highlight the need for stringent antimicrobial stewardship and infection control policies to optimize treatment outcomes, limit the emergence and spread of resistant strains, and facilitate ongoing surveillance through local antibiograms.

VFGs and antimicrobial resistance mechanisms act synergistically to enhance the pathogenicity of *P. aeruginosa*, enabling efficient adaptation, successful infection, and persistent colonization across diverse host and environmental niches (64). The *lasB* gene encodes LasB (elastase), a zinc-dependent metalloproteinase that degrades host extracellular matrix components, such as elastin and collagen, thereby promoting tissue invasion and destruction; high prevalence of *lasB* has been associated with more severe clinical outcomes in *P. aeruginosa* infections (24, 25). In the present study, all *P. aeruginosa* isolates (*n* = 175) carried at least three VFGs, a finding of considerable concern given its implications for treatment failure and severe disease. Remarkably, all isolates bore the *lasB* gene. This universal presence aligns with previous reports demonstrating consistently elevated proportions of *lasB* genes across MDR and nMDR isolates in multiple studies (45, 56). Furthermore, a high prevalence of these VFGs was observed across all MDR isolates, indicating their strong retention even under intense antibiotic pressure.

Unlike many other prevalent nosocomial pathogens, including *Staphylococcus aureus*, *Klebsiella pneumoniae*, *P. aeruginosa* exhibits a remarkable ability to evade host immune responses and inflict tissue damage via its T3SS effectors (30, 53, 54, 74). In this work, the distribution of T3SS effector genes (*exoT*, *exoS*, *exoY*, and *exoU*) was evaluated in the 175 *P. aeruginosa* isolates. The observed distribution aligns with findings previously reported in cystic fibrosis (CF) cohorts from other groups (30). Consistent with prior studies, *exoT* and *exoY* genes are present in the majority of isolates and can therefore be regarded as core genomic elements (61). ExoU, a potent phospholipase A2 that induces rapid host cell lysis and membrane disruption, is well established as a marker of severe infections, MDR phenotypes, and poorer outcomes in both respiratory and bloodstream infections (33, 34, 74, 75). ExoS possesses dual ADP-ribosyltransferase and GTPase-activating protein activities that disrupt cytoskeletal integrity, inhibit phagocytosis, and promote immune evasion (30–32). ExoS and ExoU are the most clinically relevant T3SS effectors and are frequently used as typing genes for high-risk *P. aeruginosa* clones (55, 76). Interestingly, this study identified five isolates (2.9%) harboring a rare virulotype characterized by the simultaneous presence of *exoU* and *exoS* (i.e., *exoU^+^*/*exoS^+^* virulotype), despite the well-established mutual exclusivity of *exoS* and *exoU* in most strains (Tables 2, 3, and 4). The existence of this genotype indicates the emergence of potentially hypervirulent isolates. It is necessary to conduct a comprehensive future characterization of this clinical isolate in the future to better provide information for prognosis and treatment strategies.

**TABLE 4:** The Spearman's rank correlation coefficients for the particular gene pair in 175 samples^*a*^

All (*n* = 175)		nan1	pilA	aprA	phzS	plcH	exoS	exoU	exoT	exoY	toxA
nan1	r	1.000	0.056	0.088	0.081	0.045	−0.030	0.074	0.061	0.091	−0.129
	*P*		0.465	0.247	0.288	0.558	0.692	0.332	0.426	0.233	0.091
pilA	r	0.056	1.000	−0.016	0.103	0.038	−0.014	−0.098	0.051	0.076	0.079
	*P*	0.465		0.834	0.178	0.622	0.851	0.199	0.503	0.315	0.301
aprA	r	0.088	−0.016	1.000	−0.033	−0.058	−0.001	0.050	−0.079	−0.118	−0.122
	*P*	0.247	0.834		0.663	0.445	0.995	0.513	0.300	0.119	0.109
phzS	r	0.081	0.103	−0.033	1.000	−0.142	0.030	−0.099	0.083	0.02	0.034
	*P*	0.288	0.178	0.663		0.062	0.691	0.193	0.275	0.792	0.653
plcH	r	0.045	0.038	−0.058	−0.142	1.000	0.155	0.076	0.271*	0.05	0.046
	*P*	0.558	0.622	0.445	0.062		0.040	0.315	0.000	0.508	0.544
exoS	r	−0.03	−0.014	−0.001	0.03	0.155	1.000	−0.648**	0.186	−0.01	0.023
	*P*	0.692	0.851	0.995	0.691	0.040		<0.001	0.014	0.892	0.758
exoU	r	0.074	−0.098	0.05	−0.099	0.076	−0.648**	1.000	−0.105	0.007	0.063
	*P*	0.332	0.199	0.513	0.193	0.315	<0.001		0.167	0.923	0.404
exoT	r	0.061	0.051	−0.079	0.083	0.271*	0.186	−0.105	1.000	0.085	0.079
	*P*	0.426	0.503	0.300	0.275	0.000	0.014	0.167		0.263	0.299
exoY	r	0.091	0.076	−0.118	0.02	0.05	−0.01	0.007	0.085	1.000	0.452**
	*P*	0.233	0.315	0.119	0.792	0.508	0.892	0.923	0.263		<0.001
toxA	r	−0.129	0.079	−0.122	0.034	0.046	0.023	0.063	0.079	0.452**	1.000
	*P*	0.091	0.301	0.109	0.653	0.544	0.758	0.404	0.299	<0.001	

^*a*^ Types of correlation: r > 0, positive correlation; r = 0, lack of correlation; r < 0, negative correlation; the strength of relation: <0.2, lack of relation; 0.2–0.4 (*), weak; 0.4–0.7, moderate (**); 0.7–0.9, quite strong (***); >0.9, very strong (****).

We observed that the prevalence of the *exoU* gene was comparably low in both MDR and nMDR strains (19.0% and 14.6%, respectively) (Fig. 2). Meanwhile, the samples that carry the *exoU* gene are mainly derived from sputum (*n* = 25, 86.2%). Previous studies have demonstrated that ExoU expression is associated with poorer clinical outcomes in both respiratory and bloodstream infections (77). In addition, similar low distributions of the *pilA* (encoding the major pilin subunit for type IV pilus biogenesis, twitching motility, surface attachment, and epithelial invasion) (19–21) and *nan1* (encoding neuraminidase, which enhances adhesion to host mucosal surfaces by cleaving sialic acid residues) (22) genes across MDR and nMDR strains were also reported in our work. In contrast to our finding, Mitov et al. documented a statistically significant difference in *nan1* distribution, with the gene present in 40.2% of MDR strains versus 13.2% of nMDR strains (59). Our findings suggest that, in the present cohort, *exoU*, *pilA*, and *nan1* may not serve as a dominant virulence marker differentiating MDR from nMDR strains, underscoring the need for further investigation into local epidemiology and host-pathogen dynamics.

In the MDR group, several gene pairs exhibited positive correlations, including *plcH*–*exoT*, *phzS*–*exoS*, *phzS*–*toxA*, *exoY*–*plcH*, *exoT*–*exoY*, and *exoY*–*toxA*. However, the nMDR group showed negative correlations between several pairs, notably *exoS*–*exoU*, *phz*S–*aprA*, *phzS*–*plcH*, *phzS*–*toxA*, and *exoT*–*exoU*. Although correlation-based observations do not directly inform therapeutic decision-making, which is primarily guided by isolate-specific susceptibility profiles, these correlation patterns suggest potential interactions or co-regulatory mechanisms within the virulence gene network of *P. aeruginosa* that may differ between MDR and nMDR strains (78, 79). Furthermore, the prevalence of the *phzS* gene differed significantly between MDR and nMDR isolates. The positive correlations observed between *phzS* and *aprA*, *plcH*, and *toxA* in MDR strains may contribute to enhanced overall pathogenicity. Compared with previous reports (43, 44), our findings reveal a notably high degree of genotypic diversity. Genotype XX, which was the dominant genotype among MDR isolates (35.4%) but was completely absent in nMDR strains, likely harbors a specific resistance-associated genetic background or compensatory mechanism that confers a selective advantage under multidrug pressure. Although present at lower frequencies, several additional genotypes were exclusive to MDR strains, further highlighting genotype-specific adaptations linked to resistance. Expanding the sample size and conducting longitudinal surveillance of gene prevalence and genotype dynamics in *P. aeruginosa* populations could enable the development of a predictive model based on the most discriminatory and commonly co-occurring genes in MDR versus nMDR patterns. Such a tool may facilitate early identification of high-risk strains and inform targeted infection control or therapeutic strategies.

The study has several relevant limitations. First, functional assessments of the pathogenic potential of *P. aeruginosa* strains with distinct genotypes were not analyzed, including evaluations of biofilm formation capacity and cytotoxicity. Recent studies have shown that *P. aeruginosa* harbors a large repertoire of resistance and virulence determinants, with more than 200 genes related to toxin production, biofilm formation, immune evasion, and stress adaptation (40). Future investigations should explore potential interactions among key virulence factors, such as *exoA* (encoding exotoxin A) (80) and *algD* (involved in alginate biosynthesis) (81), as well as key resistance determinants, such as β-lactam resistance genes (e.g., *blaTEM*, *blaCMY*, *blaSHV*, *blaOXA, blaVIM*, *blaIMP*, *blaNDM*) (82, 83), efflux pump systems, porin alterations (84–86), chaperone proteins (e.g., SpcU) (87), and regulators of quorum-sensing systems, such as LasR and its mutants (88). For instance, Wu et al. demonstrated that mutations in SpcU are critical for optimal ExoU secretion and cytotoxicity in highly virulent clinical isolates (87). Second, the association between specific genotypes and clinical prognosis warrants further investigation. Likewise, all samples were collected from a single center, restricting the generalizability of our findings to this specific patient population and geographic setting. Furthermore, the absence of detailed clinical characteristics of the *P. aeruginosa* infections (e.g., risk factors, management, and outcomes) precluded differentiation between true infection and colonization (42, 89). Such distinctions would provide important clinical context and help clarify whether specific genotypes are associated with pathogenicity or persistence. Future studies integrating WGS with well-annotated clinical metadata and medical imaging will be essential to clarify the clinical significance of these genotypes and to support evidence-based antimicrobial stewardship strategies.

## MATERIALS AND METHODS

### Biological samples and identification

A total of 175 non-duplicate clinical isolates of *P. aeruginosa* collected from the medical laboratory of NYCH were included in the study. The samples were received between July and November 2025. Samples were stored at 4°C for no more than 2 days prior to processing.

Clinical specimens were processed according to standard microbiological diagnostic procedures in our unit. Initial identification of *P. aeruginosa* cultured from clinical samples was based on the colony morphology and microscopic characteristics in Petri dishes with blood agar (Oxoid, USA), chocolate agar (Autobio, China), and Mac-Conkey agar (BD Difco, USA) plates at aerobic conditions for 18 h at 37°C. Definitive identification was performed using the MALDI-TOF mass spectrometry system (Autof ms1000, Zhengzhou, China). After identification, all isolates were cryopreserved in 40% glycerol at −80°C for further analysis. All *P. aeruginosa* strains included in this study were obtained from distinct clinical specimens derived from different patients.

### Antimicrobial susceptibility test

AST was conducted on 175 isolates using the following steps: an inoculum of each bacterial isolate was prepared by diluting an overnight culture of each isolate to a 0.5 McFarland standard (1.5 × 10^8^ cfu/mL) in sterile Mueller–Hinton broth (Oxoid, USA); minimum inhibitory concentration for each antimicrobial agent was determined using the VITEK-2 Compact automated system with GN 335 cards (bioMérieux, Craponne, France). Any equivocal results were verified by the Kirby-Bauer agar disk diffusion method (90). Results were interpreted as susceptible, intermediate, or resistant according to the 2025 Clinical and Laboratory Standards Institute guidelines and breakpoints specific to *P. aeruginosa*. The following 16 antibiotics from eight classes were evaluated: Aminoglycosides (AGs, tobramycin, amikacin), Penicillins (PCNs, piperacillin), Carbapenems (CBPs, meropenem, imipenem), Cephalosporins (CEPs, ceftazidime, cefepime), Fluoroquinolones (FQs, ciprofloxacin, levofloxacin), Monobactams (MBs, aztreonam), Polymyxins (colistin B, colistin E), and β-lactams/β-lactamase inhibitors (BL/BLI, piperacillin/tazobactam, ceftazidime/avibactam, imipenem/relebactam, and ticarcillin/clavulanic acid). According to established recommendations by the European Center for Disease Prevention and Control (3), MDR *P. aeruginosa* was defined as acquired non-susceptibility to at least one agent in three or more antimicrobial categories. In contrast, isolates not meeting these criteria were classified as nMDR (non-susceptible to no more than two antimicrobial classes) (91). *P. aeruginosa* ATCC 27853 was used as the quality control strain.

### Preparation of bacterial genomic DNA

Each confirmed *P. aeruginosa* isolate was streaked from single colonies and grown overnight in Luria-Bertani broth with constant shaking (180 rpm) at 37°C for 18 h. The overnight cultures were centrifuged at 12,000 × *g* for 5 min at 4°C, and the supernatants were discarded. Genomic DNA was isolated and purified using the Genomic Mini Kit (Qiagen, Germany) according to the manufacturer’s protocol. DNA concentration and purity were assessed using a NanoDrop 2000 spectrophotometer (Thermo Fisher Scientific, USA) by measuring absorbance at 260 and 280 nm. All the DNA samples were stored at −20°C until used for PCR analysis.

### VFGs detection

The prevalence of VFGs was determined by PCR, with a separate reaction performed for each gene. Briefly, each 20 µL PCR reaction contained 10 µL of 2× FastTaq PCR SuperMix (Servicebio, Wuhan, China), 0.4 µM each of forward and reverse primers specific to the target gene, 2 µL of template genomic DNA from each *P. aeruginosa* isolate, and nuclease-free water to complete the volume. Amplification was carried out in an Applied Biosystems 2720 thermal cycler (Thermo Fisher Scientific, USA) under the following amplification conditions: 95°C, 5 min; followed by 30 cycles of 95°C for 30 s, 57°C for 30 s, and 72°C for 30–50 s; and a final extension at 72°C for 7 min. The resulting PCR product was separated by electrophoresis on 1.0% agarose gels pre-stained with ethidium bromide in 1× TAE buffer using a Bio-Rad system (Feldkirchen, Germany) and visualized under the GS-800 (TM) Calibrated Densitometer (Bio-Rad, CA, USA). Almost all primer sequences used for the amplification of the VFGs were widely validated and applied in previous studies (30, 43–45, 59–63) (Table S1). Genomic DNA from *P. aeruginosa* reference strains ATCC 27853 and PAO1 isolate served as PCR positive controls for the investigation of particular genes. Initial positive PCR results were confirmed by DNA sequencing, which did not reveal significant mismatches affecting detection. Subsequent positive results were therefore considered validated based on PCR alone.

### Statistical analysis

Spearman’s rank correlation coefficients were calculated to evaluate the relationships between the presence of specific genes within the nMDR and MDR groups. Differences in the prevalence of individual genes and genotypes between nMDR and MDR strains were assessed using the chi-square test or Fisher’s exact test. All data were analyzed using RStudio (version 4.4.0) packages. A *P* value less than 0.05 was regarded as statistically significant (*P < 0.05, **P < 0.01, ***P < 0.001).

## Data Availability

The data presented in this study are available on request from the corresponding author.

## SUPPLEMENTAL MATERIAL

Supplemental material spectrum.00973-26-s0001.pdf Fig. S1 to S4; Tables S1 to S12.

ASM does not own the copyrights to Supplemental Material that may be linked to, or accessed through, an article. The authors have granted ASM a non-exclusive, world-wide license to publish the Supplemental Material files. Please contact the corresponding author directly for reuse.
