# Supplementary material for: Prevalence and genotypic distribution of virulence factor genes and antibiotic susceptibility profiles in clinical Pseudomonas aeruginosa isolates from a public hospital in Nanyang, China
Source: Microbiol Spectr. 2026 Jun 29;14(8):e00973-26. doi: 10.1128/spectrum.00973-26 (PMC13435649; doi:10.1128/spectrum.00973-26)
Supplement: Supplementary file 1 [file spectrum.00973-26-s0001.pdf]

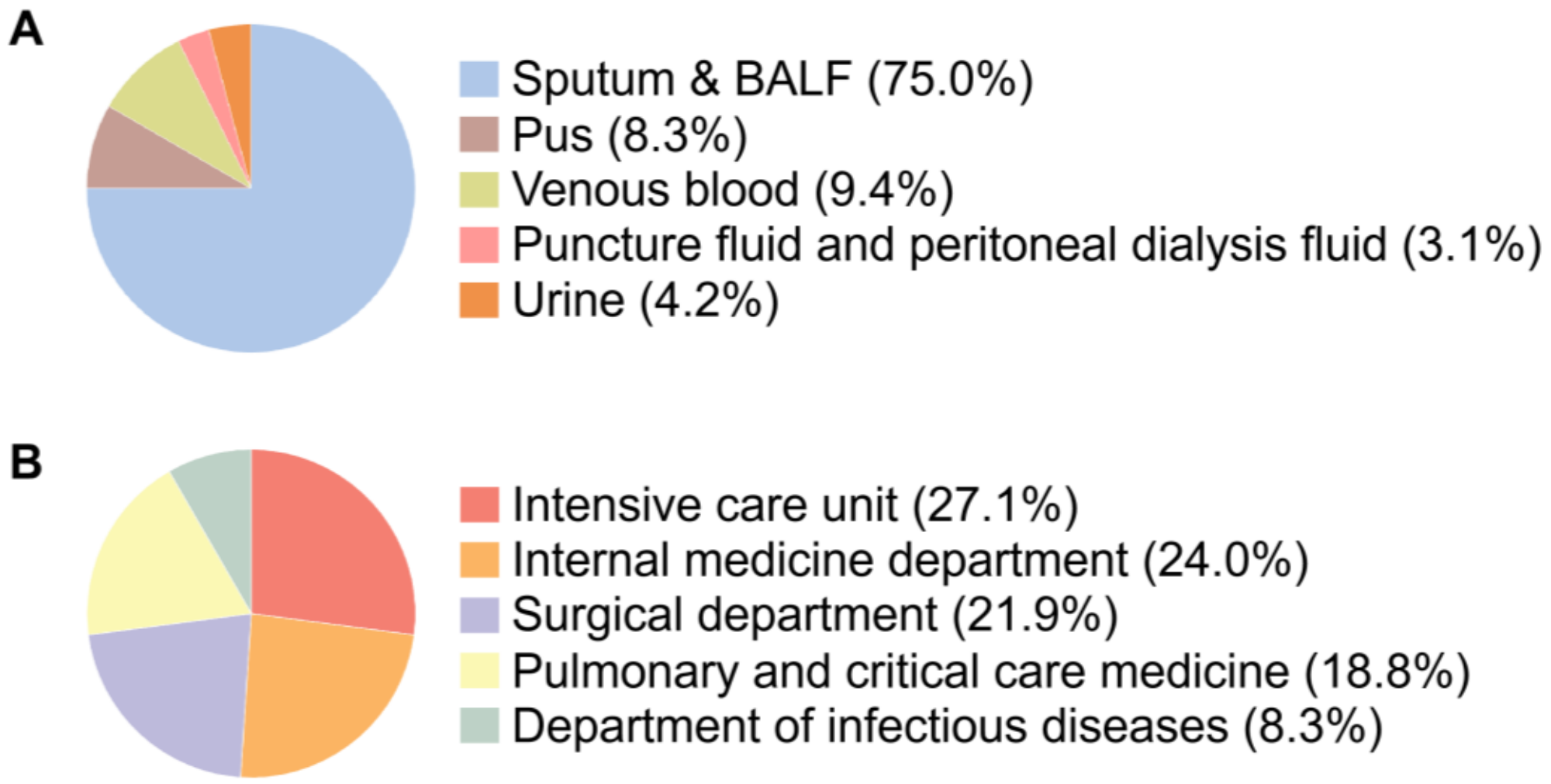

Fig. S1 The specimen types (A) and departments (B) of 96 nMDR *P. aeruginosa* included in this study.

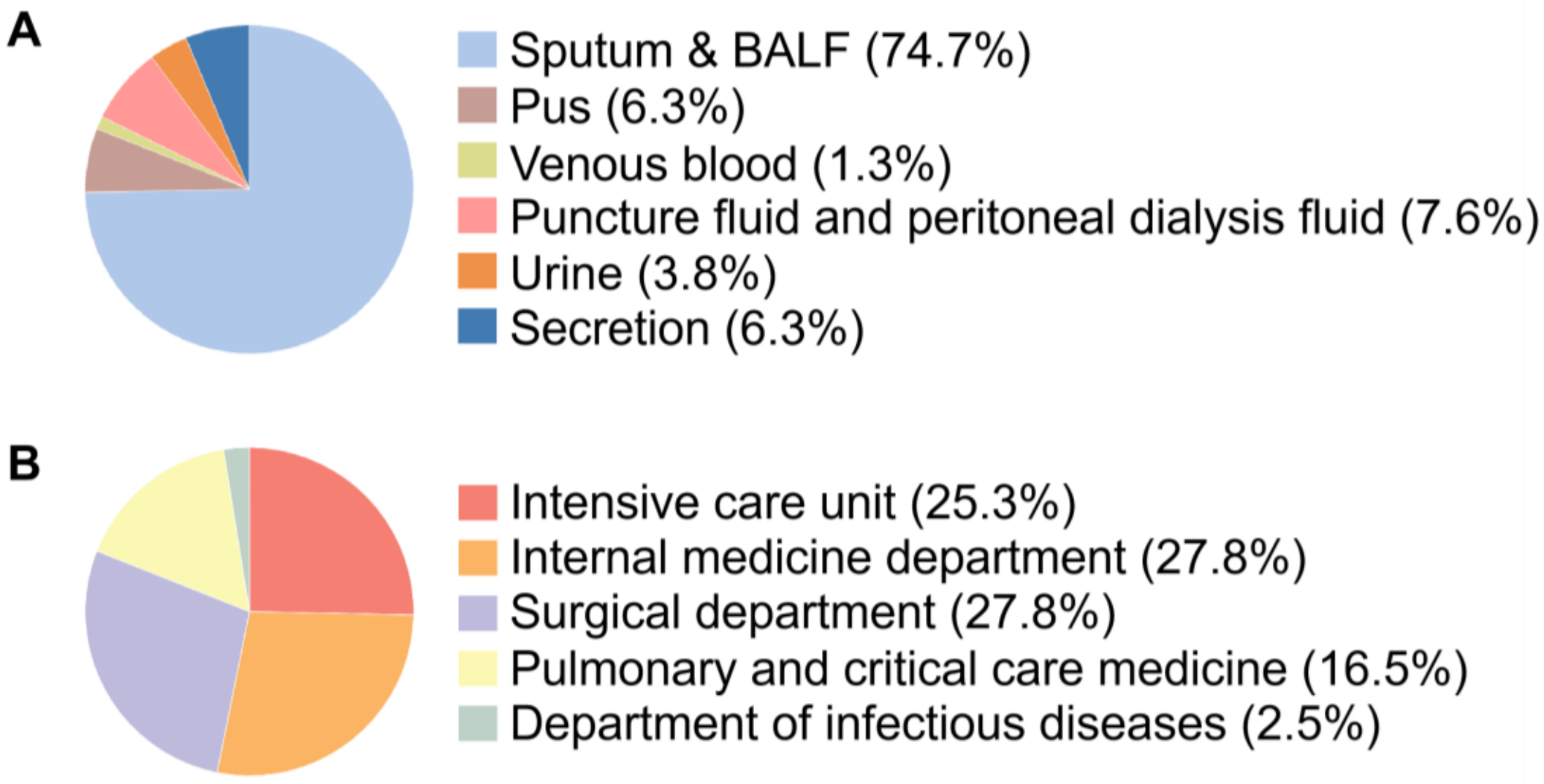

Fig. S2 The specimen types (A) and departments (B) of 79 MDR *P. aeruginosa* included in this study.

Table S1 Primers used in this study.

Genes	Sequences (5′ to 3′)	Size (bp)	Reference
nan1	F: ATGAATACTTATTTTGATAT<br>R: CTAAATCCATGCTCTGACCC	1316	(1, 2)
exoU	F: ATGCATATCCAATCGTTG<br>R: TCATGTGAACTCCTTATT	2000	(3, 4)
exoS	F: CTTGAAGGGACTCGACAAGG<br>R: TTCAGGTCCGCGTAGTGAAT	500	(1)
toxA	F: GGTAACCAGCTCAGCCACAT<br>R: TGATGTCCAGGTCATGCTTC	350	(1, 3, 5)
aprA	F: TGTCCAGCAATTCTCTTGC<br>R: CGTTTTCCACGGTGACC	1000	(3)
exoY	F: GTCTCTACAGGATCAGCCGC<br>R: CGTCGCTGTGGTGAAACATC	330	(6)
exoT	F: TGCGGTAATGGACAAGGTCG<br>R: AACAGGGTGGTTATCGTGCC	450	(6)
plcH	F: GAAGCCATGGGCTACTTCAA<br>R: AGAGTGACGAGGAGCGGTAG	300	(3, 7, 8)
lasB	F: GGAATGAACGAAGCGTTCTC<br>R: GGTCCAGTAGTAGCGGTTGG	300	(1, 7, 8)
oprI	F: ATGAACAACGTTCTGAAATTCTCTGC<br>R: CTTGCGGCTGGCTTTTTCCAG	252	(1, 9)
oprL	F: ATGGAAATGCTGAAATTCGGC<br>R: CTTCTTCAGCTCGACGCGACG	500	(1)
gyrB	F: TGGGAACAGGTCTACCACCA<br>R: CAGACCGCCTTCGTACTTGA	250	(6)
pilA	F: GGCTTTACCTTGATCG<br>R: ATCACAACCTTTCGGAG	400	This study
phzS	F: ATCGATATCCTCATCGCC<br>R: TCCTCGATACGCTCCA	480	This study

Table S2 The results of ASTs of *P. aeruginosa* strains in this study (The MDR strains are marked in bold).

Clinical strain	BL/BLI	FQs	MBs	CEPs	CBPs	PCNs	AGs	Polymyxins
**PA-1**	**R**	**R**	**R**	**R**	S	S	S	S
PA-2	**R**	S	**R**	S	S	S	S	S
PA-3	S	**R**	S	S	S	S	S	S
**PA-4**	**R**	**R**	S	S	**R**	S	S	S
PA-5	S	S	**R**	S	S	S	S	S
**PA-6**	**R**	S	**R**	**R**	S	S	S	S
PA-7	S	**R**	S	S	S	S	S	S
PA-8	**R**	S	**R**	S	S	S	S	S
PA-9	S	S	**R**	S	S	S	S	S
**PA-10**	**R**	**R**	**R**	S	S	S	S	S
PA-11	S	**R**	S	S	S	S	S	S
PA-12	S	**R**	**R**	S	S	S	S	S
PA-13	**R**	S	**R**	S	S	S	S	S
PA-14	S	**R**	S	S	S	S	S	S
PA-15	S	S	**R**	S	S	S	S	S
**PA-16**	**R**	S	**R**	**R**	S	S	S	S
PA-17	S	**R**	S	S	S	S	S	S
**PA-18**	**R**	**R**	**R**	**R**	**R**	S	S	S
PA-19	S	**R**	S	S	S	S	**R**	S
PA-20	S	**R**	S	S	S	S	S	S
**PA-21**	**R**	S	**R**	**R**	S	S	S	S
**PA-22**	**R**	**R**	**R**	**R**	S	**R**	S	S
PA-23	S	S	**R**	S	S	S	S	S
PA-24	S	**R**	S	S	S	S	S	S
PA-25	S	S	S	S	**R**	S	S	S
**PA-26**	**R**	**R**	**R**	S	S	S	S	S
PA-27	S	**R**	S	S	**R**	S	S	S
PA-28	S	**R**	S	S	S	S	S	S
**PA-29**	**R**	**R**	**R**	**R**	**R**	S	**R**	S
**PA-30**	S	**R**	S	**R**	S	S	**R**	S
PA-31	S	**R**	S	S	S	S	S	S
**PA-32**	**R**	**R**	S	S	S	**R**	S	S
**PA-33**	**R**	S	**R**	**R**	**R**	**R**	S	S
PA-34	S	S	**R**	**R**	S	S	S	S
**PA-35**	**R**	**R**	**R**	S	S	S	S	S
PA-36	S	**R**	S	S	S	S	S	S
PA-37	S	S	**R**	S	S	S	S	S
PA-38	**R**	S	**R**	S	S	S	S	S
**PA-39**	**R**	**R**	**R**	**R**	**R**	**R**	**R**	S
PA-40	S	S	**R**	S	S	S	S	S

**PA-41**	**R**	**R**	**R**	S	S	S	S	S
PA-42	S	S	**R**	S	S	S	S	S
**PA-43**	**R**	**R**	**R**	**R**	S	S	S	S
PA-44	**R**	S	**R**	S	S	S	S	S
PA-45	**R**	**R**	S	S	S	S	S	S
PA-46	S	S	**R**	S	S	S	S	S
**PA-47**	**R**	**R**	**R**	S	**R**	S	S	S
**PA-48**	**R**	S	**R**	**R**	S	S	S	S
PA-49	**R**	**R**	S	S	S	S	S	S
PA-50	S	S	**R**	S	S	S	S	S
**PA-51**	**R**	**R**	**R**	**R**	**R**	S	S	S
PA-52	S	S	**R**	S	S	S	S	S
**PA-53**	**R**	**R**	**R**	S	S	S	S	S
**PA-54**	**R**	**R**	S	S	**R**	S	S	S
**PA-55**	**R**	S	**R**	S	**R**	S	S	S
PA-56	S	**R**	S	S	S	S	S	S
**PA-57**	**R**	**R**	**R**	S	**R**	S	S	S
PA-58	S	**R**	S	S	**R**	S	S	S
**PA-59**	**R**	**R**	S	**R**	S	**R**	S	S
**PA-60**	**R**	S	**R**	**R**	S	S	S	S
PA-61	**R**	S	**R**	S	S	S	S	S
PA-62	S	**R**	S	S	S	S	S	S
**PA-63**	**R**	S	S	**R**	**R**	S	**R**	S
PA-64	S	**R**	S	S	S	S	S	S
**PA-65**	**R**	S	S	**R**	S	S	**R**	S
**PA-66**	**R**	**R**	S	**R**	**R**	**R**	**R**	S
PA-67	**R**	S	**R**	S	S	S	S	S
**PA-68**	**R**	**R**	**R**	S	**R**	S	S	S
PA-69	S	S	**R**	S	S	S	S	S
PA-70	**R**	S	S	S	S	S	S	S
**PA-71**	**R**	S	**R**	S	**R**	S	S	S
PA-72	S	**R**	S	S	S	S	S	S
**PA-73**	S	**R**	S	**R**	**R**	S	**R**	S
**PA-74**	**R**	**R**	S	**R**	**R**	S	S	S
PA-75	S	**R**	S	S	S	S	S	S
**PA-76**	**R**	**R**	**R**	S	**R**	S	S	S
PA-77	S	S	**R**	S	S	S	S	S
PA-78	S	S	**R**	S	S	S	S	S
PA-79	S	S	**R**	S	S	S	S	S
PA-80	S	**R**	S	S	S	S	S	S
PA-81	S	S	**R**	S	S	S	S	S
**PA-82**	**R**	**R**	S	S	**R**	S	S	S
PA-83	S	S	**R**	S	S	S	S	S
**PA-84**	**R**	**R**	**R**	**R**	S	S	S	S

PA-85	S	S	**R**	S	S	S	S	S
PA-86	**R**	S	**R**	S	S	S	S	S
PA-87	**R**	S	**R**	S	S	S	S	S
PA-88	S	**R**	S	S	S	S	S	S
**PA-89**	**R**	**R**	**R**	**R**	**R**	**R**	S	S
**PA-90**	**R**	**R**	**R**	S	S	S	S	S
**PA-91**	**R**	**R**	**R**	S	S	S	S	S
**PA-92**	**R**	**R**	**R**	**R**	S	S	S	S
**PA-93**	**R**	S	**R**	**R**	S	S	S	S
PA-94	S	**R**	S	S	**R**	S	S	S
**PA-95**	**R**	S	**R**	**R**	S	S	S	S
PA-96	S	**R**	S	S	S	S	S	S
**PA-97**	**R**	**R**	**R**	S	S	S	S	S
PA-98	S	**R**	S	S	S	S	S	S
**PA-99**	**R**	**R**	S	S	**R**	S	S	S
**PA-100**	**R**	**R**	**R**	**R**	**R**	**R**	S	S
PA-101	**R**	**R**	S	S	S	S	S	S
**PA-102**	**R**	**R**	S	**R**	S	**R**	**R**	S
PA-103	**R**	S	**R**	S	S	S	S	S
**PA-104**	**R**	**R**	**R**	**R**	S	**R**	S	S
**PA-105**	**R**	**R**	**R**	**R**	S	**R**	S	S
PA-106	**R**	S	**R**	S	S	S	S	S
**PA-107**	**R**	S	**R**	S	**R**	S	S	S
**PA-108**	**R**	**R**	**R**	S	S	S	S	S
PA-109	S	S	**R**	S	S	S	S	S
**PA-110**	**R**	S	**R**	**R**	**R**	S	**R**	S
**PA-111**	**R**	**R**	**R**	**R**	**R**	**R**	S	S
PA-112	**R**	**R**	S	S	S	S	S	S
**PA-113**	**R**	**R**	**R**	**R**	**R**	**R**	S	S
PA-114	S	**R**	S	S	S	S	S	S
**PA-115**	**R**	**R**	**R**	**R**	S	**R**	S	S
PA-116	**R**	S	**R**	S	S	S	S	S
**PA-117**	**R**	**R**	**R**	**R**	**R**	**R**	S	S
PA-118	S	**R**	S	S	S	S	S	S
PA-119	S	S	**R**	S	S	S	S	S
**PA-120**	**R**	**R**	S	**R**	S	**R**	S	S
PA-121	S	**R**	S	S	S	S	S	S
PA-122	S	S	S	S	S	S	S	S
PA-123	S	**R**	S	S	S	**R**	S	S
**PA-124**	**R**	**R**	S	**R**	**R**	**R**	**R**	S
PA-125	S	**R**	S	S	S	S	S	S
**PA-126**	**R**	S	**R**	S	**R**	S	S	S
**PA-127**	**R**	**R**	**R**	**R**	**R**	**R**	**R**	S
**PA-128**	**R**	S	**R**	S	**R**	S	S	S

PA-129	**R**	S	S	**R**	S	S	S	S
PA-130	S	**R**	S	S	S	S	S	S
PA-131	S	**R**	S	S	S	S	S	S
PA-132	S	S	**R**	S	S	S	S	S
PA-133	S	S	**R**	S	S	S	S	S
PA-134	**R**	S	**R**	S	S	S	S	S
PA-135	**R**	S	**R**	S	S	S	S	S
PA-136	S	**R**	S	S	S	S	S	S
PA-137	**R**	S	**R**	S	S	S	S	S
PA-138	S	**R**	S	S	S	S	S	S
PA-139	**R**	S	S	**R**	S	S	S	S
**PA-140**	**R**	**R**	**R**	**R**	**R**	S	**R**	S
PA-141	S	**R**	S	S	S	S	S	S
**PA-142**	**R**	S	S	**R**	**R**	S	S	S
**PA-143**	**R**	**R**	**R**	S	**R**	S	S	S
PA-144	S	**R**	S	S	S	S	S	S
PA-145	S	**R**	S	S	S	S	S	S
**PA-146**	**R**	**R**	**R**	S	S	S	S	S
PA-147	S	**R**	S	S	S	S	S	S
**PA-148**	**R**	**R**	**R**	**R**	S	**R**	S	S
**PA-149**	**R**	**R**	S	S	**R**	S	S	S
PA-150	S	**R**	S	S	S	S	S	S
PA-151	S	**R**	S	S	S	S	S	S
**PA-152**	**R**	**R**	**R**	**R**	S	**R**	S	S
PA-153	S	**R**	S	S	S	S	S	S
**PA-154**	**R**	**R**	S	S	**R**	S	S	S
PA-155	**R**	**R**	S	S	S	S	S	S
PA-156	S	S	**R**	S	S	S	S	S
PA-157	S	**R**	S	S	S	S	S	S
**PA-158**	**R**	S	**R**	**R**	S	S	S	S
PA-159	S	S	**R**	S	S	S	S	S
PA-160	**R**	S	**R**	S	S	S	S	S
**PA-161**	**R**	**R**	S	S	S	**R**	S	S
PA-162	S	**R**	S	S	**R**	S	S	S
PA-163	S	**R**	S	S	**R**	S	S	S
PA-164	S	S	**R**	S	S	S	S	S
**PA-165**	**R**	**R**	**R**	S	S	S	S	S
**PA-166**	**R**	**R**	**R**	**R**	**R**	S	**R**	S
PA-167	S	**R**	S	S	S	S	S	S
**PA-168**	**R**	**R**	**R**	**R**	S	**R**	S	S
**PA-169**	**R**	**R**	S	**R**	S	**R**	S	S
**PA-170**	**R**	**R**	**R**	S	S	S	S	S
PA-171	S	**R**	S	S	S	S	S	S
**PA-172**	**R**	**R**	S	S	**R**	S	S	S

**PA-173**	**R**	**R**	**R**	**R**	**R**	**R**	S	S
**PA-174**	**R**	**R**	**R**	**R**	**R**	**R**	S	S
**PA-175**	**R**	**R**	S	S	**R**	S	S	S

Key: AGs = Aminoglycosides, PCNs = Penicillins, CBPs = Carbapenems, CEPs = Cephalosporins, FQs = Fluoroquinolones, MBs = Monobactams, BL/BLI = β-lactams/ β-lactamase inhibitors.

Table S3 Overall resistance rates of the *P. aeruginosa* isolates to antibiotics in this study.

Antibiotics	Percentage of resistance
BL/BLI	101 (57.7%)
FQs	111 (63.4%)
MBs	99 (56.6%)
CEPs	50 (28.6%)
CBPs	46 (26.3%)
PCNs	26 (14.9%)
AGs	14 (8.0%)
Polymyxins	0 (0.0%)

Key: AGs = Aminoglycosides, PCNs = Penicillins, CBPs = Carbapenems, CEPs = Cephalosporins, FQs = Fluoroquinolones, MBs = Monobactams, BL/BLI = β-lactams/β-lactamase inhibitors.

Table S4 The Spearman's rank correlation coefficients among the resistance patterns of different antibiotic to 175 samples. (types of correlation: r > 0 positive correlation, r = 0 lack of correlation, r < 0 negative correlation; the strength of relation: < 0.2 - lack of relation, 0.2 - 0.4 - weak, 0.4 - 0.7 -moderate, 0.7 - 0.9 - quite strong, > 0.9 - very strong).

**All (n=175)**		**PCNs**	**BL/BLI**	**CEPs**	**AGs**	**FQs**	**MBs**	**CBPs**
**PCNs**	**r**	1.000	0.325*	0.554**	0.173	0.284*	0.074	0.189
	p		< 0.001	< 0.001	0.022	< 0.001	0.329	0.012
**BL/BLI**	**r**	0.325*	1.000	0.465**	0.124	- 0.002	0.370*	0.327*
	p	< 0.001		< 0.001	0.101	0.984	< 0.001	< 0.001
**CEPs**	**r**	0.554**	0.465**	1.000	0.420**	0.060	0.197	0.255*
	p	< 0.001	< 0.001		< 0.001	0.43	0.009	0.001
**AGs**	**r**	0.173	0.124	0.420**	1.000	0.093	- 0.082	0.302*
	p	0.022	0.101	< 0.001		0.222	0.283	<0.001
**FQs**	**r**	0.284*	- 0.002	0.060	0.093	1.000	- 0.474**	0.184
	p	< 0.001	0.984	0.43	0.222		< 0.001	0.015
**MBs**	**r**	0.074	0.370*	0.197	- 0.082	- 0.474**	1.000	- 0.001
	p	0.329	< 0.001	0.009	0.283	< 0.001		0.994
**CBPs**	**r**	0.189	0.327*	0.255*	0.302*	0.184	- 0.001	1.000
	p	0.012	< 0.001	0.001	< 0.001	0.015	0.994	

Key: AGs = Aminoglycosides, PCNs = Penicillins, CBPs = Carbapenems, CEPs = Cephalosporins, FQs = Fluoroquinolones, MBs = Monobactams, BL/BLI = β-lactams/ β-lactamase inhibitors.

Table S5 The Spearman's rank correlation coefficients among the resistance patterns of different antibiotic to 79 MDR samples. (types of correlation: r > 0 positive correlation, r = 0 lack of correlation, r < 0 negative correlation; the strength of relation: < 0.2 - lack of relation, 0.2 - 0.4 - weak, 0.4 - 0.7 - moderate, 0.7 - 0.9 - quite strong, > 0.9 - very strong).

**MDR (n = 79)**		**PCNs**	**BL/BLI**	**CEPs**	**AGs**	**FQs**	**MBs**	**CBPs**
**PCNs**	**r**	1.000	0.11	0.451**	0.065	0.305*	- 0.063	- 0.036
	p		0.336	< 0.001	0.569	0.006	0.581	0.754
**BL/BLI**	**r**	0.11	1.000	- 0.133	- 0.363*	- 0.088	0.259*	0.002
	p	0.336		0.243	0.001	0.443	0.021	0.986
**CEPs**	**r**	0.451**	- 0.133	1.000	0.366*	- 0.141	0.063	- 0.093
	p	< 0.001	0.243		0.001	0.216	0.584	0.416
**AGs**	**r**	0.065	- 0.363*	0.366*	1.000	- 0.003	- 0.257*	0.233*
	p	0.569	0.001	0.001		0.978	0.022	0.038
**FQs**	**r**	0.305*	- 0.088	- 0.141	- 0.003	1.000	- 0.136	0.007
	p	0.006	0.443	0.216	0.978		0.234	0.952
**MBs**	**r**	- 0.063	0.259*	0.063	- 0.257*	- 0.136	1.000	- 0.162
	p	0.581	0.021	0.584	0.022	0.234		0.155
**CBPs**	**r**	- 0.036	0.002	- 0.093	0.233*	0.007	- 0.162	1.000
	p	0.754	0.986	0.416	0.038	0.952	0.155	

Key: AGs = Aminoglycosides, PCNs = Penicillins, CBPs = Carbapenems, CEPs = Cephalosporins, FQs = Fluoroquinolones, MBs = Monobactams, BL/BLI = β-lactams/ β-lactamase inhibitors.

Table S6 The Spearman's rank correlation coefficients among the resistance patterns of different antibiotic to 96 nMDR samples. (types of correlation: r > 0 positive correlation, r = 0 lack of correlation, r < 0 negative correlation; the strength of relation: < 0.2 - lack of relation, 0.2 - 0.4 - weak, 0.4 - 0.7 -moderate, 0.7 - 0.9 - quite strong, > 0.9 - very strong).

**nMDR (n = 96)**		**PCNs**	**BL/BLI**	**CEPs**	**AGs**	**FQs**	**MBs**	**CBPs**
**PCNs**	**r**	1.000	- 0.059	- 0.018	- 0.011	0.098	- 0.09	- 0.026
	p		0.566	0.859	0.919	0.340	0.381	0.798
**BL/BLI**	**r**	- 0.059	1.000	0.173	- 0.059	- 0.361*	0.267*	- 0.149
	p	0.566		0.092	0.566	< 0.001	0.009	0.147
**CEPs**	**r**	- 0.018	0.173	1.000	- 0.018	- 0.187	- 0.038	- 0.046
	p	0.859	0.092		0.859	0.068	0.715	0.654
**AGs**	**r**	- 0.011	- 0.059	- 0.018	1.000	0.098	- 0.09	- 0.026
	p	0.919	0.566	0.859		0.340	0.381	0.798
**FQs**	**r**	0.098	- 0.361*	- 0.187	0.098	1.000	- 0.877***	0.162
	p	0.340	< 0.001	0.068	0.340		< 0.001	0.116
**MBs**	**r**	- 0.09	0.267*	- 0.038	- 0.09	- 0.877***	1.000	- 0.228*
	p	0.381	0.009	0.715	0.381	< 0.001		0.026
**CBPs**	**r**	- 0.026	- 0.149	- 0.046	- 0.026	0.162	- 0.228*	1.000
	p	0.798	0.147	0.654	0.798	0.116	0.026	

Key: AGs = Aminoglycosides, PCNs = Penicillins, CBPs = Carbapenems, CEPs = Cephalosporins, FQs = Fluoroquinolones, MBs = Monobactams, BL/BLI = β-lactams/ β-lactamase inhibitors.

Table S7 Antibiotics resistance patterns of *P. aeruginosa* isolates.

Phenotypic pattern	Number of MDR isolates	Number of nMDR isolates
PCNs BL/BLI CEPs AGs FQs MBs CBPs	2 (2.5%)	0 (0.0%)
PCNs BL/BLI CEPs AGs FQs CBPs	2 (2.5%)	0 (0.0%)
PCNs BL/BLI CEPs AGs FQs	1 (1.3%)	0 (0.0%)
PCNs BL/BLI CEPs FQs MBs CBPs	7 (8.9%)	0 (0.0%)
PCNs BL/BLI CEPs FQs MBs	7 (8.9%)	0 (0.0%)
PCNs BL/BLI CEPs FQs	3 (3.8%)	0 (0.0%)
PCNs BL/BLI CEPs MBs CBPs	1 (1.3%)	0 (0.0%)
PCNs BL/BLI FQs	2 (2.5%)	0 (0.0%)
PCNs FQs	0 (0.0%)	1 (1.0%)
BL/BLI CEPs AGs FQs MBs CBPs	3 (3.8%)	0 (0.0%)
BL/BLI CEPs AGs MBs CBPs	1 (1.3%)	0 (0.0%)
BL/BLI CEPs AGs CBPs	1 (1.3%)	0 (0.0%)
BL/BLI CEPs AGs	1 (1.3%)	0 (0.0%)
BL/BLI CEPs FQs MBs CBPs	2 (2.5%)	0 (0.0%)
BL/BLI CEPs FQs MBs	4 (5.1%)	0 (0.0%)
BL/BLI CEPs FQs CBPs	1 (1.3%)	0 (0.0%)
BL/BLI CEPs MBs	8 (10.1%)	0 (0.0%)
BL/BLI CEPs CBPs	1 (1.3%)	0 (0.0%)
BL/BLI CEPs	0 (0.0%)	2 (2.1%)
BL/BLI FQs MBs CBPs	5 (6.3%)	0 (0.0%)
BL/BLI FQs MBs	12 (15.2%)	0 (0.0%)
BL/BLI FQs CBPs	8 (10.1%)	0 (0.0%)
BL/BLI FQs	0 (0.0%)	5 (5.2%)
BL/BLI MBs CBPs	5 (6.3%)	0 (0.0%)
BL/BLI MBs	0 (0.0%)	16 (16.7%)
BL/BLI	0 (0.0%)	1 (1.0%)
CEPs AGs FQs CBPs	1 (1.3%)	0 (0.0%)
CEPs AGs FQs	1 (1.3%)	0 (0.0%)
CEPs MBs	0 (0.0%)	1 (1.0%)
AGs FQs	0 (0.0%)	1 (1.0%)
FQs MBs	0 (0.0%)	1 (1.0%)
FQs CBPs	0 (0.0%)	5 (5.2%)
FQs	0 (0.0%)	37 (38.5%)
MBs	0 (0.0%)	24 (25.0%)
CBPs	0 (0.0%)	1 (1.0%)

Pan-Susceptible	0 (0.0%)	1 (1.0%)

Key: AGs = Aminoglycosides, PCNs = Penicillins, CBPs = Carbapenems, CEPs = Cephalosporins, FQs = Fluoroquinolones, MBs = Monobactams, BL/BLI = β-lactams/β-lactamase inhibitors.

Table S8 Distribution of VFGs in *P. aeruginosa* isolates.

Genes	Number of *P. aeruginosa* with VFGs (n = 175)	Number of MDR isolates (n = 79)	Number of nMDR isolates (n = 96)
pilA	8 (4.6%)	4 (5.1%）	4 (4.2%)
nan1	12 (6.9%)	8 (10.1%)	4 (4.2%)
exoU	29 (16.6%)	15 (19.0%)	14 (14.6%)
phzS	69 (39.4%)	47 (59.5%)	22 (22.9%)
exoS	136 (77.7%)	62 (78.5%)	74 (77.1%)
toxA	155 (88.6%)	71 (89.9%)	84 (87.5%)
aprA	157 (89.7%)	74 (93.7%)	83 (86.5%)
exoY	156 (89.1%)	75 (95.0%)	81 (84.4%)
exoT	166 (94.9%)	77 (97.5%)	89 (92.7%)
plcH	170 (97.1%)	77 (97.5%)	93 (96.9%)
lasB	175 (100.0%)	79 (100.0%)	96 (100.0%)
oprI	175 (100.0%)	79 (100.0%)	96 (100.0%)
oprL	175 (100.0%)	79 (100.0%)	96 (100.0%)
gyrB	175 (100.0%)	79 (100.0%)	96 (100.0%)

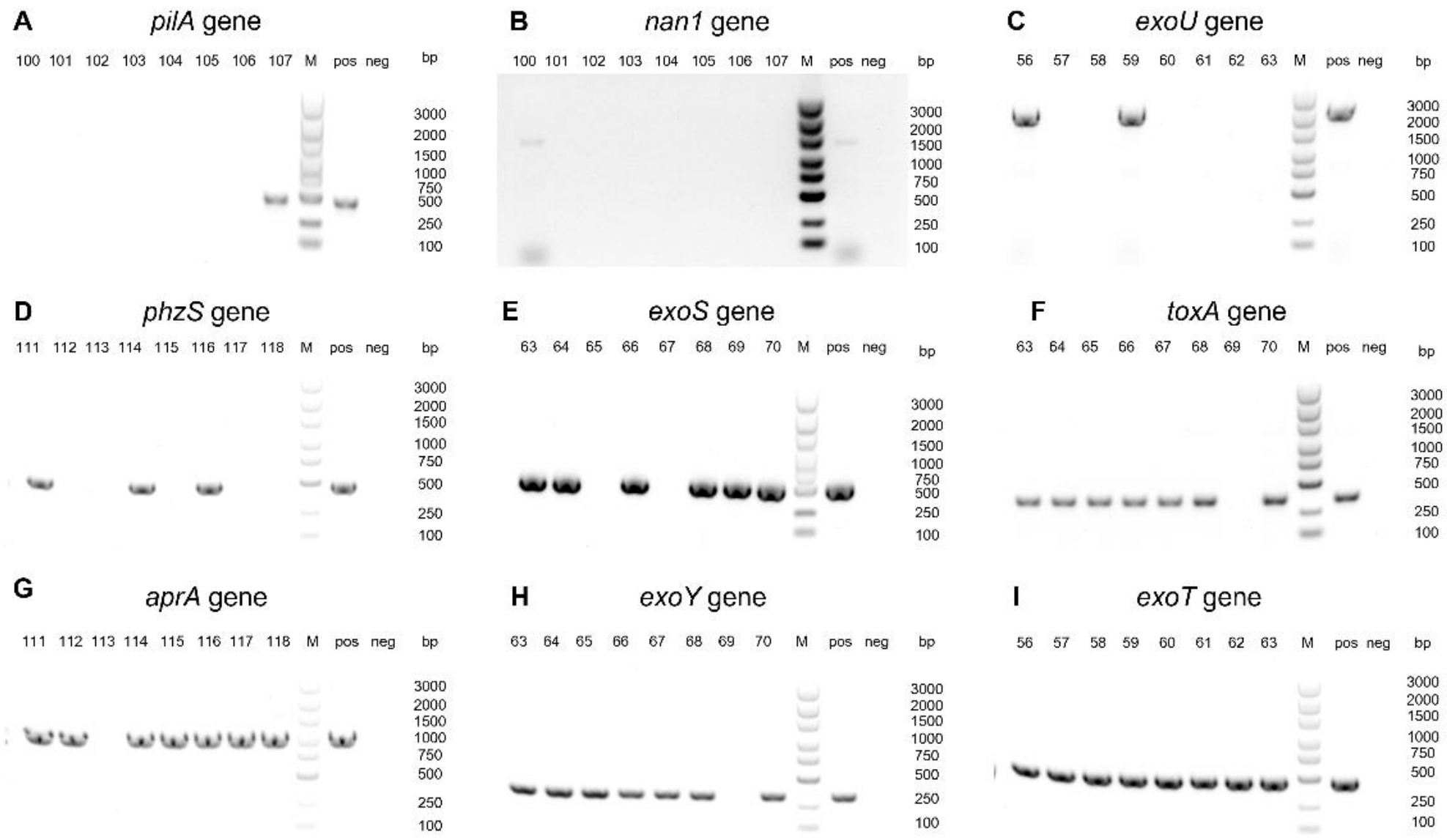

Fig. S3. The gel electrophoresis display of tested genes (A) *pilA* (B) *nan1* (C) *exoU* (D) *phzS* (E) *exoS* (F) *toxA* (G) *aprA* (H) *exoY* (I) *exoT* in representative clinical strains (pos: positive control, neg: negative control, M: marker).

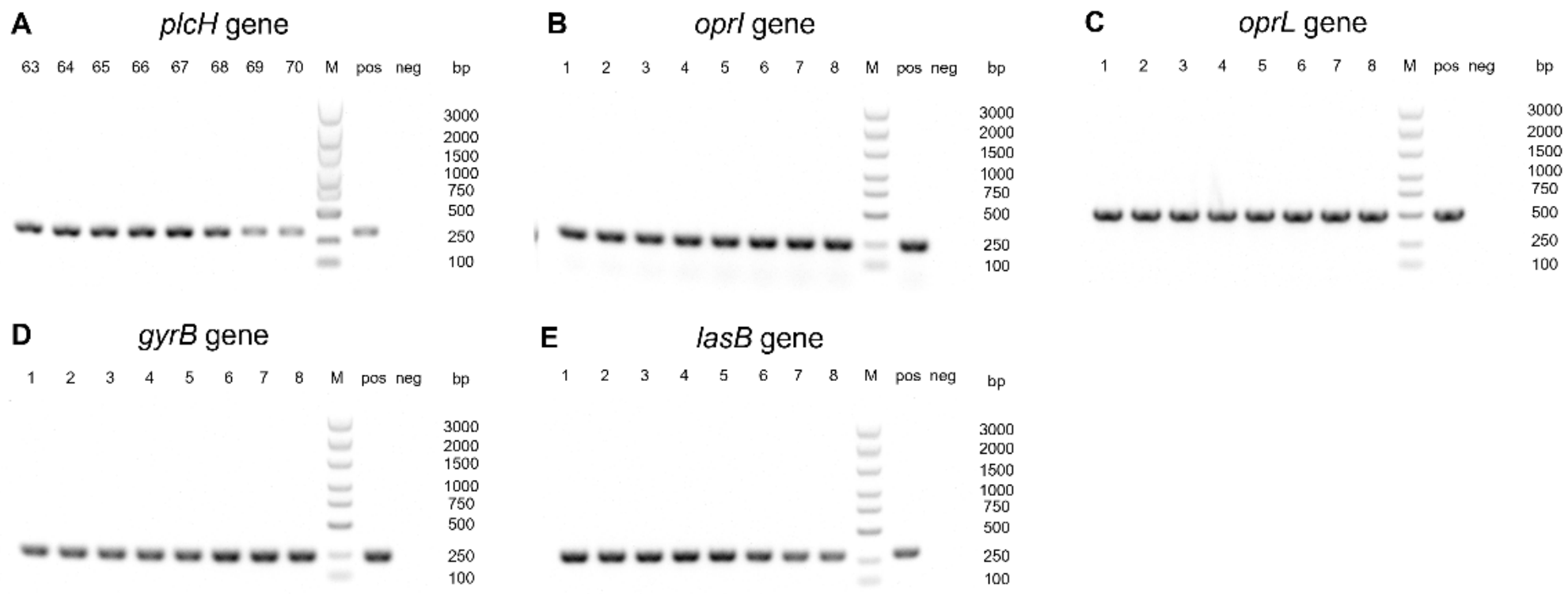

Fig. S4. The gel electrophoresis display of tested genes (A) *plcH* (B) *oprI* (C) *oprL* (D) *gyrB* (E) *lasB* in representative clinical strains (pos: positive control, neg: negative control, M: marker).

Table S9 The detailed incidence of the detected VFGs amongst the MDR strains.

Clinical strain	*nan1*	*pilA*	*aprA*	*phzS*	*plcH*	*exoS*	*exoU*	*exoT*	*exoY*	*toxA*	*lasB*	*oprL*	*gyrB*	*oprI*
PA-1	+	-	+	+	+	+	-	+	+	+	+	+	+	+
PA-4	-	-	+	+	+	+	-	+	+	+	+	+	+	+
PA-6	-	-	+	-	+	+	-	+	+	-	+	+	+	+
PA-10	-	-	+	+	+	+	-	+	-	+	+	+	+	+
PA-16	-	-	+	-	+	-	+	+	-	-	+	+	+	+
PA-18	-	-	+	-	+	+	-	+	+	+	+	+	+	+
PA-21	-	-	+	-	+	+	-	+	+	-	+	+	+	+
PA-22	+	-	+	+	+	+	-	+	+	+	+	+	+	+
PA-26	-	-	+	+	+	+	-	+	+	+	+	+	+	+
PA-29	+	-	+	+	+	-	+	+	+	+	+	+	+	+
PA-30	-	-	+	+	+	+	-	+	+	+	+	+	+	+
PA-32	-	-	+	+	+	+	-	+	+	+	+	+	+	+
PA-33	-	-	+	-	+	+	-	+	-	-	+	+	+	+
PA-35	-	-	+	+	+	+	-	+	+	+	+	+	+	+
PA-39	-	-	+	+	+	-	+	+	+	+	+	+	+	+
PA-41	-	-	+	+	+	+	-	+	+	+	+	+	+	+
PA-43	+	-	+	+	+	+	-	+	+	+	+	+	+	+
PA-47	-	-	+	-	+	+	-	+	+	+	+	+	+	+
PA-48	-	-	+	-	+	+	-	+	+	+	+	+	+	+
PA-51	-	-	+	-	+	+	-	+	+	+	+	+	+	+
PA-53	+	-	+	+	+	-	-	+	+	-	+	+	+	+
PA-54	-	-	+	+	+	+	-	+	+	+	+	+	+	+
PA-55	-	-	+	+	+	+	-	+	+	+	+	+	+	+
PA-57	-	-	+	+	+	-	-	+	+	+	+	+	+	+
PA-59	-	-	+	-	+	-	+	+	+	+	+	+	+	+
PA-60	-	-	+	+	+	+	-	+	+	+	+	+	+	+

PA-63	+	+	+	+	+	+	-	+	+	+	+	+	+	+
PA-65	-	-	+	-	+	-	-	+	+	+	+	+	+	+
PA-66	-	-	+	-	+	+	-	+	+	+	+	+	+	+
PA-68	-	-	+	+	+	+	-	+	+	+	+	+	+	+
PA-71	-	-	+	+	-	+	-	+	+	+	+	+	+	+
PA-73	-	-	+	+	+	+	-	+	+	+	+	+	+	+
PA-74	-	-	+	+	+	+	-	+	+	+	+	+	+	+
PA-76	-	-	+	+	+	+	-	+	+	+	+	+	+	+
PA-82	-	-	+	+	+	+	-	+	+	+	+	+	+	+
PA-84	-	-	+	-	+	-	-	+	+	-	+	+	+	+
PA-89	-	-	+	-	+	+	-	+	+	+	+	+	+	+
PA-90	-	-	+	-	+	-	-	+	+	+	+	+	+	+
PA-91	-	-	+	+	+	+	-	+	+	+	+	+	+	+
PA-92	-	-	+	-	+	+	-	+	+	+	+	+	+	+
PA-93	-	-	+	+	+	+	+	+	+	+	+	+	+	+
PA-95	-	-	+	+	+	+	+	+	+	+	+	+	+	+
PA-97	-	-	+	-	-	-	-	-	-	+	+	+	+	+
PA-99	+	-	+	-	+	+	+	+	+	+	+	+	+	+
PA-100	+	-	+	-	+	+	-	+	+	-	+	+	+	+
PA-102	-	-	+	-	+	+	-	+	+	-	+	+	+	+
PA-104	-	-	+	-	+	+	-	-	+	+	+	+	+	+
PA-105	-	-	-	-	+	+	-	+	+	+	+	+	+	+
PA-107	-	+	+	+	+	+	-	+	+	+	+	+	+	+
PA-108	-	-	+	+	+	+	-	+	+	+	+	+	+	+
PA-110	-	-	+	+	+	+	-	+	+	+	+	+	+	+
PA-111	-	+	+	+	+	+	-	+	+	+	+	+	+	+
PA-113	-	-	-	-	+	+	-	+	+	+	+	+	+	+
PA-115	-	-	+	-	+	-	+	+	+	+	+	+	+	+
PA-117	-	-	+	-	+	-	+	+	+	+	+	+	+	+

PA-120	-	-	-	-	+	-	+	+	+	+	+	+	+	+
PA-124	-	-	+	+	+	+	-	+	+	+	+	+	+	+
PA-126	-	-	-	+	+	+	-	+	+	+	+	+	+	+
PA-127	-	-	+	+	+	+	-	+	+	+	+	+	+	+
PA-128	-	-	+	+	+	+	-	+	+	+	+	+	+	+
PA-140	-	-	+	+	+	+	-	+	+	+	+	+	+	+
PA-142	-	-	+	+	+	-	-	+	+	+	+	+	+	+
PA-143	-	-	+	-	+	+	-	+	+	+	+	+	+	+
PA-146	-	-	-	-	+	+	-	+	+	+	+	+	+	+
PA-148	-	-	+	-	+	-	+	+	+	+	+	+	+	+
PA-149	-	-	+	+	+	+	-	+	+	+	+	+	+	+
PA-152	-	-	+	+	+	+	-	+	+	+	+	+	+	+
PA-154	-	-	+	-	+	+	-	+	+	+	+	+	+	+
PA-158	-	-	+	+	+	+	-	+	+	+	+	+	+	+
PA-161	-	-	+	+	+	+	-	+	+	+	+	+	+	+
PA-165	-	-	+	+	+	+	+	+	+	+	+	+	+	+
PA-166	-	-	+	+	+	+	-	+	+	+	+	+	+	+
PA-168	-	+	+	+	+	+	-	+	+	+	+	+	+	+
PA-169	-	-	+	-	+	-	+	+	+	+	+	+	+	+
PA-170	-	-	+	-	+	+	+	+	+	+	+	+	+	+
PA-172	-	-	+	-	+	+	-	+	+	+	+	+	+	+
PA-173	-	-	+	+	+	+	-	+	+	+	+	+	+	+
PA-174	-	-	+	+	+	-	+	+	+	+	+	+	+	+
PA-175	-	-	+	+	+	+	-	+	+	+	+	+	+	+

Table S10 The detailed incidence of the detected VFGs amongst the nMDR strains.

Clinical strain	*nan1*	*pilA*	*aprA*	*phzS*	*plcH*	*exoS*	*exoU*	*exoT*	*exoY*	*toxA*	*lasB*	*oprL*	*gyrB*	*oprI*
PA-2	-	-	+	-	+	+	-	+	+	+	+	+	+	+
PA-3	-	-	+	+	+	+	-	+	-	-	+	+	+	+
PA-5	-	-	+	-	+	-	+	+	+	+	+	+	+	+
PA-7	-	-	+	-	+	+	-	+	+	-	+	+	+	+
PA-8	-	-	+	+	+	+	-	+	-	+	+	+	+	+
PA-9	+	-	+	-	+	-	+	+	+	+	+	+	+	+
PA-11	-	-	+	-	+	+	-	+	+	+	+	+	+	+
PA-12	-	-	+	-	+	+	-	+	-	-	+	+	+	+
PA-13	-	-	+	-	+	-	+	+	+	+	+	+	+	+
PA-14	-	-	+	-	+	-	+	+	+	+	+	+	+	+
PA-15	-	-	+	-	+	-	+	+	-	+	+	+	+	+
PA-17	-	-	+	-	+	+	-	+	-	-	+	+	+	+
PA-19	-	-	+	-	+	-	+	+	+	+	+	+	+	+
PA-20	-	-	+	-	+	+	-	+	-	+	+	+	+	+
PA-23	-	-	+	-	+	+	-	+	+	+	+	+	+	+
PA-24	-	-	+	-	+	-	+	+	+	+	+	+	+	+
PA-25	-	-	+	-	+	+	-	+	+	+	+	+	+	+
PA-27	-	-	+	-	+	-	+	+	+	+	+	+	+	+
PA-28	-	-	+	-	+	+	-	+	+	+	+	+	+	+
PA-31	-	-	+	-	+	+	-	+	-	+	+	+	+	+
PA-34	-	-	+	-	+	+	-	+	-	-	+	+	+	+
PA-36	-	-	+	-	+	+	-	+	+	+	+	+	+	+
PA-37	-	-	+	-	+	+	-	+	+	+	+	+	+	+
PA-38	-	-	+	-	+	+	-	+	+	+	+	+	+	+
PA-40	-	-	+	-	+	+	-	+	+	+	+	+	+	+
PA-42	-	-	+	-	+	+	-	+	+	+	+	+	+	+

PA-44	-	-	+	-	+	+	-	+	+	+	+	+	+	+
PA-45	-	-	+	-	+	+	-	+	+	+	+	+	+	+
PA-46	-	-	+	-	+	+	-	+	+	+	+	+	+	+
PA-49	-	-	+	-	+	+	-	+	+	+	+	+	+	+
PA-50	-	-	+	-	+	+	-	+	+	+	+	+	+	+
PA-52	-	-	+	+	+	+	-	+	+	-	+	+	+	+
PA-56	-	-	+	-	+	-	+	+	+	+	+	+	+	+
PA-58	-	-	+	+	-	+	-	+	+	+	+	+	+	+
PA-61	-	-	+	-	+	-	-	+	+	+	+	+	+	+
PA-62	-	-	+	-	+	+	-	+	+	+	+	+	+	+
PA-64	-	-	+	-	+	+	-	+	±	+	+	+	+	+
PA-67	-	-	+	+	+	-	+	-	±	+	+	+	+	+
PA-69	-	-	+	+	+	+	-	+	-	-	+	+	+	+
PA-70	-	-	+	-	+	+	-	+	±	+	+	+	+	+
PA-72	-	+	+	+	+	-	-	+	+	+	+	+	+	+
PA-75	-	-	+	-	+	-	+	+	+	+	+	+	+	+
PA-77	-	-	+	+	-	-	-	-	+	-	+	+	+	+
PA-78	-	-	+	-	+	+	-	-	+	+	+	+	+	+
PA-79	-	-	+	-	+	-	+	-	+	+	+	+	+	+
PA-80	-	-	+	-	+	+	-	-	+	+	+	+	+	+
PA-81	-	-	+	-	+	+	-	+	+	+	+	+	+	+
PA-83	-	-	+	-	+	+	-	+	+	+	+	+	+	+
PA-85	-	-	+	-	+	+	-	+	+	+	+	+	+	+
PA-86	+	-	+	-	+	+	-	+	+	+	+	+	+	+
PA-87	-	-	+	-	+	+	-	+	+	+	+	+	+	+
PA-88	+	-	+	-	+	+	-	+	+	+	+	+	+	+
PA-94	-	-	+	-	+	+	-	+	+	+	+	+	+	+
PA-96	-	-	+	+	+	-	-	+	+	+	+	+	+	+
PA-98	-	-	+	-	+	+	-	+	+	+	+	+	+	+

PA-101	-	-	+	-	+	+	-	+	+	+	+	+	+	+
PA-103	-	-	+	-	+	-	+	-	-	-	+	+	+	+
PA-106	-	-	-	-	+	+	-	+	+	+	+	+	+	+
PA-109	-	-	-	-	+	+	-	+	+	+	+	+	+	+
PA-112	-	-	+	-	+	+	-	+	-	+	+	+	+	+
PA-114	-	-	+	+	-	-	-	+	+	+	+	+	+	+
PA-116	-	-	+	+	+	+	-	+	-	+	+	+	+	+
PA-118	-	-	+	-	+	+	-	+	+	+	+	+	+	+
PA-119	-	-	-	+	+	+	-	+	+	+	+	+	+	+
PA-121	-	-	-	+	+	+	-	+	+	+	+	+	+	+
PA-122	-	-	-	+	+	+	-	+	+	+	+	+	+	+
PA-123	-	-	-	-	+	+	-	+	+	+	+	+	+	+
PA-125	-	-	+	-	+	+	-	+	+	+	+	+	+	+
PA-129	-	-	-	+	+	+	-	+	+	+	+	+	+	+
PA-130	-	-	+	-	+	+	-	+	+	+	+	+	+	+
PA-131	-	-	+	-	+	+	-	+	+	+	+	+	+	+
PA-132	-	-	+	-	+	+	-	+	+	+	+	+	+	+
PA-133	-	+	+	-	+	+	-	+	+	+	+	+	+	+
PA-134	-	-	+	-	+	+	-	+	+	+	+	+	+	+
PA-135	-	-	+	-	+	+	-	-	+	+	+	+	+	+
PA-136	-	-	+	+	+	-	-	+	+	+	+	+	+	+
PA-137	-	-	-	+	+	-	-	+	+	+	+	+	+	+
PA-138	-	+	+	-	+	+	-	+	+	+	+	+	+	+
PA-139	-	-	+	-	+	+	-	+	+	+	+	+	+	+
PA-141	-	-	+	-	+	+	-	+	+	+	+	+	+	+
PA-144	-	-	+	+	+	+	-	+	-	+	+	+	+	+
PA-145	-	-	+	-	+	+	-	+	+	+	+	+	+	+
PA-147	-	-	-	+	+	-	+	+	+	+	+	+	+	+
PA-150	-	-	+	-	+	+	-	+	+	+	+	+	+	+

PA-151	-	-	-	-	+	+	-	+	+	+	+	+	+	+
PA-153	-	-	+	-	+	+	-	+	+	+	+	+	+	+
PA-155	-	-	+	-	+	+	-	+	+	+	+	+	+	+
PA-156	-	-	+	-	+	+	-	+	+	+	+	+	+	+
PA-157	-	-	+	-	+	+	-	+	+	+	+	+	+	+
PA-159	-	-	+	+	+	+	-	+	-	-	+	+	+	+
PA-160	-	-	-	+	+	+	-	+	+	+	+	+	+	+
PA-162	-	+	-	-	+	-	-	+	+	+	+	+	+	+
PA-163	-	-	-	-	+	+	-	+	+	+	+	+	+	+
PA-164	+	-	+	+	+	+	-	+	+	-	+	+	+	+
PA-167	-	-	+	-	+	+	-	+	-	-	+	+	+	+
PA-171	-	-	+	-	+	+	-	+	+	+	+	+	+	+

Table S11 The departments of XDR *P. aeruginosa*.

Unit origins	Number of XDR isolates
**Surgical department**	7 (50.0%)
**Internal medicine department**	3 (21.4%)
**Intensive care unit**	4 (28.6%)

Table S12 The specimen types of XDR *P. aeruginosa*.

**Specimen types**	**Number of XDR isolates**
**Sputum & BALF**	8 (57.1%)
**Secretion**	3 (21.4%)
**Pus**	1 (7.1%)
**Puncture fluid and peritoneal dialysis fluid**	1 (7.1%)
**Urine**	1 (7.1%)

REFERENCES

1. Adenipekun EO, Akinleye EF, Tewogbade OA, Iwalokun BA. 2023. Detection of virulence genes and multidrug resistance in *Pseudomonas aeruginosa* clinical isolates from a public hospital in Lagos, Nigeria. Scientific African 22: e01950.

2. Mitov I, Strateva T, Markova B. 2010. Prevalence of virulence genes among bulgarian nosocomial and cystic fibrosis isolates of *pseudomonas aeruginosa*. Braz J Microbiol 41: 588-95.

3. Wang X, Gao K, Pan B, Wang B, Song Y, Guo W. 2025. The virulence trait and genotype distribution amongst the *Pseudomonas aeruginosa* clinical strains. BMC Microbiol 25: 82.

4. Petit SM, Lavenir R, Colinon-Dupuich C, Boukerb AM, Cholley P, Bertrand X, Freney J, Doléans-Jordheim A, Nazaret S, Laurent F, Cournoyer B. 2013. Lagooning of wastewaters favors dissemination of clinically relevant *Pseudomonas aeruginosa*. Res Microbiol 164: 856-66.

5. Bogiel T, Depka D, Rzepka M, Kwiecińska-Piróg J, Gospodarek-Komkowska E. 2021. Prevalence of the Genes Associated with Biofilm and Toxins Synthesis amongst the *Pseudomonas aeruginosa* Clinical Strains. Antibiotics (Basel) 10: 241.

6. Nolasco-Romero CG, Prado-Galbarro FJ, Jimenez-Juarez RN, Gomez-Ramirez U, Cancino-Díaz JC, López-Marceliano B, Apodaca MR, Aguayo-Romero MA, Rodea GE, Pichardo-Villalon L, Parra-Ortega I, Santos FS, Moreno-Galván M, Velázquez-Guadarrama N. 2024. The exoS, exoT, exoU and exoY Virulotypes of the Type 3 Secretion System in Multidrug Resistant *Pseudomonas aeruginosa* as a Death Risk Factor in Pediatric Patients. Pathogens 13: 1030.

7. Bogiel T, Depka D, Kruszewski S, Rutkowska A, Kanarek P, Rzepka M, Leitão JH, Deptuła A, Gospodarek-Komkowska E. 2023. Comparison of Virulence-Factor-Encoding Genes and Genotype Distribution amongst Clinical *Pseudomonas aeruginosa* Strains. Int J Mol Sci 24: 1269.

8. Lanotte P, Watt S, Mereghetti L, Dartiguelongue N, Rastegar-Lari A, Goudeau A, Quentin R. 2004. Genetic features of *Pseudomonas aeruginosa* isolates from cystic fibrosis patients compared with those of isolates from other origins. J Med Microbiol

53: 73-81.
9. De Vos D, Lim A, Jr., De Vos P, Sarniguet A, Kersters K, Cornelis P. 1993. Detection of the outer membrane lipoprotein I and its gene in fluorescent and non-fluorescent pseudomonas: implications for taxonomy and diagnosis. J Gen Microbiol 139: 2215-23.
